# Neuronal cholesterol metabolism increases dendritic outgrowth and synaptic markers via a concerted action of GGTase-I and Trk

**DOI:** 10.1038/srep30928

**Published:** 2016-08-05

**Authors:** Miguel Moutinho, Maria João Nunes, Jorge C. Correia, Maria João Gama, Margarida Castro-Caldas, Angel Cedazo-Minguez, Cecília M. P. Rodrigues, Ingemar Björkhem, Jorge L Ruas, Elsa Rodrigues

**Affiliations:** 1 Research Institute for Medicines (iMed.ULisboa), Faculty of Pharmacy, Universidade de Lisboa, Portugal, Av. Prof. Gama Pinto, 1649-003 Lisboa, Portugal; 2 Department of Physiology and Pharmacology, Molecular and Cellular Exercise Physiology, Karolinska Institutet, 17177 Stockholm, Sweden; 3 Department of Biochemistry and Human Biology, Faculty of Pharmacy, Universidade de Lisboa, Av. Prof. Gama Pinto, 1649-003 Lisboa, Portugal; 4 Department of Life Sciences, Faculty of Science and Technology, Universidade NOVA de Lisboa, 2829-516 Caparica, Portugal; 5 Department of Neurobiology, Care Sciences and Society, Karolinska Institutet-Alzheimer’s Disease Research Center, Novum, Stockholm, Sweden; 6 Department of Laboratory Medicine, Division of Clinical Chemistry, Karolinska Institutet, Karolinska University Hospital, Huddinge, Sweden

## Abstract

Cholesterol 24-hydroxylase (CYP46A1) is responsible for brain cholesterol elimination and therefore plays a crucial role in the control of brain cholesterol homeostasis. Altered CYP46A1 expression has been associated with several neurodegenerative diseases and changes in cognition. Since CYP46A1 activates small guanosine triphosphate-binding proteins (sGTPases), we hypothesized that CYP46A1 might be affecting neuronal development and function by activating tropomyosin-related kinase (Trk) receptors and promoting geranylgeranyl transferase-I (GGTase-I) prenylation activity. Our results show that CYP46A1 triggers an increase in neuronal dendritic outgrowth and dendritic protrusion density, and elicits an increase of synaptic proteins in the crude synaptosomal fraction. Strikingly, all of these effects are abolished by pharmacological inhibition of GGTase-I activity. Furthermore, CYP46A1 increases Trk phosphorylation, its interaction with GGTase-I, and the activity of GGTase-I, which is crucial for the enhanced dendritic outgrowth. Cholesterol supplementation studies indicate that cholesterol reduction by CYP46A1 is the necessary trigger for these effects. These results were confirmed *in vivo*, with a significant increase of p-Trk, pre- and postsynaptic proteins, Rac1, and decreased cholesterol levels, in crude synaptosomal fractions prepared from CYP46A1 transgenic mouse cortex. This work describes the molecular mechanisms by which neuronal cholesterol metabolism effectively modulates neuronal outgrowth and synaptic markers.

Dysfunction in brain cholesterol homeostasis has been widely associated with neurodegenerative disorders and cognitive decline^1^. The neuronal-specific cytochrome P450 cholesterol 24-hydroxylase (CYP46A1) is a major player in brain cholesterol homeostasis. This enzyme is responsible for the conversion of cholesterol into 24S-hydroxycholesterol (24OHC), the major pathway for brain cholesterol elimination^2^,^3^,^4^,^5^. CYP46A1 polymorphisms have been associated with higher risk of Alzheimer´s disease (AD)^6^,^7^ and faster course of cognitive deterioration in late life^8^,^9^. Interestingly, in mouse models of AD, delivery of CYP46A1 to the brain by a recombinant virus vector reduces amyloid pathology before or after the onset of amyloid plaques^10^. Conversely, delivery of short hairpin RNA directed against the mouse Cyp46a1 mRNA leads to an increase of cholesterol levels in neurons, followed by amyloidogenesis and neuronal loss^11^. Additionally, cholesterol 24-hydroxylase modulates brain cholesterol synthesis by a feedback mechanism that regulates the mevalonate pathway. In fact, the mevalonate pathway is suppressed in the brain of Cyp46a1 knockout (Cyp46a1^−/−^) mice^12^, and activated in homozygous CYP46A1 transgenic (C46-HA) mouse brain, maintaining cholesterol at physiological levels^13^. Interestingly, Cyp46a1^−/−^ mice exhibit severe deficiencies in spatial, associative, and motor learning, and in hippocampal long-term potentiation. In contrast, C46-HA mice exhibit an improved cognitive performance, when compared to wild type (WT) mice^12^,^13^. However, although the mechanisms by which CYP46A1, the major enzyme of brain cholesterol metabolism, affects neuronal function are not clear, the differences in cognitive function are likely due to changes in the supply of isoprenoid intermediates of the mevalonate pathway^5^,^12^,^14^, which are essential for protein prenylation. Indeed, we have recently demonstrated that increased CYP46A1 expression induces prenylation, namely geranylgeranylation and activity of small guanosine triphosphate-binding proteins (sGTPases) in neuronal cells^15^. The activity of sGTPases has been extensively related to neuronal development and function. It has been recently described that geranylgeranyl transferase I (GGTase-I), the enzyme responsible for geranylgeranylation of sGTPases, is activated by brain-derived neurothrophic factor (BDNF), through direct protein-protein interaction with the tropomyosin-related kinase B (TrkB) receptors, which seems to be crucial for BDNF-induced dendritic development^16^. On the other hand, it has been reported that cholesterol loss leads to TrkB auto-activation^17^.

Taking these data into consideration, we hypothesized that there is a crosstalk between CYP46A1-induced sGTPase prenylation and Trk activation, which could mediate the beneficial effects of cholesterol 24-hydroxylase activity on neuronal development and function.

Herein we report that neuronal cholesterol metabolism, mediated by CYP46A1, promotes neuronal outgrowth and increases synaptic markers in a GGTase-I-dependent fashion. We show that the increased GGTase-I activity that results from the activation of the Trk-GGTase-I axis by CYP46A1 is crucial for the increment of dendritic outgrowth. This axis, however, does not appear to be essential for the increase in dendritic protrusion density mediated by CYP46A1. Nevertheless, although acting as independent mechanisms, both activation of Trk due to cholesterol loss, and GGTase-I activity are essential for the full effect of CYP46A1 on these synaptic structures.

This study describes the molecular mechanisms by which CYP46A1 modulates neuronal outgrowth and function, highlighting the cholesterol 24-hydroxylase as an attractive drug target in the physiological and pathological context of cognitive decline and brain disorders.

## Results

### CYP46A1 induces dendritic outgrowth in a GGTase-I dependent manner

**Figure 1:**
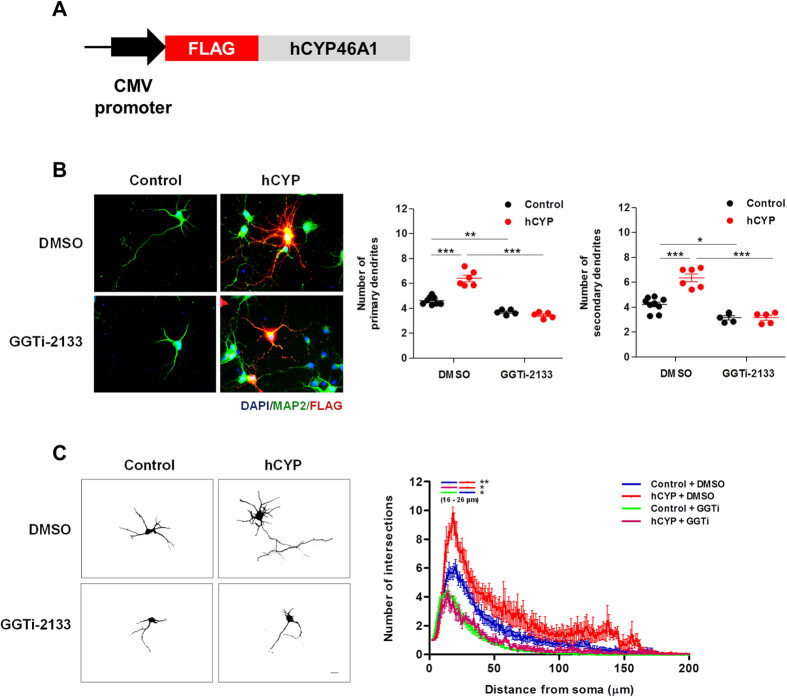
CYP46A1 increases the number of primary and secondary dendrites and overall dendritic arbor complexity in a GGTase-I dependent manner. Primary cultures of rat cortical neurons kept for 4 days *in vitro* (4 DIV) were transfected with Control or hCYP vector and maintained for 48 hours. 24 hours after transfection, cells were incubated with 100 nM of GGTi-2133 or vehicle (DMSO). Neurons were stained with DAPI, and immunostained with anti-FLAG and -MAP2 antibodies for dendritic outgrowth analysis at 6 DIV. (**A**) Schematic structure of pCMV-FLAG-hCYP46A1 plasmid encoding for the enzyme CYP46A1 tagged with the FLAG epitope (**B**) Quantitation of primary and secondary dendrites of neurons in all experimental conditions. Images are representative of merged DAPI (blue), FLAG (red) and MAP2 (green) staining in Control and hCYP transfected neurons treated with DMSO or GGTi-2133. Scale bar: 20 μm. Data represents mean values ± SEM from at least three independent experiments and is expressed as number of dendrites. Statistical analysis was performed by two-way ANOVA followed by Tukey post-hoc test (*p < 0.05 **p < 0.01 ***p < 0.001). (**C**) Quantitation of dendritic arbor complexity. Sholl analysis was performed in neurons transfected with Control or hCYP vector and treated with DMSO or GGTi-2133. Images are representative of 6 DIV neurons in each condition. Scale bar: 20 μm. Data represents mean values ± SEM from at least three independent experiments and is plotted as number of intersections versus distance (μm) to soma. Statistical analysis was performed by two-way ANOVA and post-hoc Tukey test (*p < 0.05 **p < 0.01).

We have recently shown that increased expression and activity of CYP46A1 induces the activation of the mevalonate pathway, which consequently leads to increased prenylation and activation of sGTPases, including Rho family proteins^15^. Since in neurons, Rho GTPases regulate several features of dendritic and axonal outgrowth during development and regeneration, mainly through their effects on the cytoskeleton, we started by evaluating the role of CYP46A1 on dendritic outgrowth in primary cultures of rat cortical neurons. Neurons kept for 4 days *in vitro* (DIV) were transfected with a control plasmid harboring a CMV promoter, pCMV (Control), or the same plasmid encoding for the FLAG-tagged human CYP46A1, pCMV-FLAG-hCYP46A1 (hCYP) (Fig. 1A) and maintained for 48 hours. CYP46A1 protein and mRNA endogenous levels as well as the ectopic expression of FLAG-tagged CYP46A1 and the levels of its product, 24OHC, in culture media are shown in Supplementary Fig. 1. As previously shown, CYP46A1 mRNA and protein levels increase during neuronal maturation *in vitro* (Supplementary Fig. 1A,B). Moreover, transfection of hCYP vector robustly increased CYP46A1 protein and 24OHC levels in neurons (Supplementary Fig. 1C,D). These cells were incubated with 100 nM GGTi-2133, a geranylgeranyl transferase-I (GGTase-I) inhibitor, or vehicle (DMSO) in the last 24 hours after transfection. Subsequently, neurons were fixed, stained with DAPI (nuclear stain) and immunostained with anti-FLAG and -MAP2 antibodies for dendritic outgrowth analysis at 6 DIV (Fig. 1B,C). Our results show that the number of both primary and secondary dendrites was significantly increased in hCYP-transfected neurons. However, inhibiting prenylation not only decreased the number of dendrites but it also impaired the CYP46A1-dependent effect (Fig. 1B) (Two-way ANOVA *p* < 0.001, Tukey HSD post hoc test). Results from Sholl analysis confirmed a significant increase in dendritic arbor complexity after hCYP transfection, while the GGTi-2133 treatment decreased dendritic arborization and abolished the CYP46A1 effect (Fig. 1C). Particularly, CYP46A1 significantly increased the number of intersections at 16–25 μm from the soma when compared to control neurons. On the other hand, GGTi-2133 treatment decreased the basal number of intersections and inhibited the increase promoted by CYP46A1, respectively (two-way ANOVA *p* < 0.05, Tukey HSD post hoc test).

Since we have previously shown that 24OHC has a different effect on the activation of sGTPases than CYP46A1 ectopic expression^15^, and to determine if the CYP46A1-mediated effect on neuronal dendritic outgrowth is not dependent on the increased production of this oxysterol, we have treated 5 DIV neurons for 24 h with vehicle or 10 μM 24OHC. Quantitation of primary dentrites and dendritic arbor complexity was performed as previously described. Our results show that 24OHC has the opposite effect of CYP46A1 (Supplementary Fig. 2), since it significantly decreases dendritic outgrowth *in vitro*.

Overall, our results show that CYP46A1 ectopic expression promotes neuronal dendritic outgrowth in a GGTase-I dependent manner.

### GGTase-I is necessary for the CYP46A1-mediated increase of dendritic protrusions and synaptic protein enrichment

**Figure 2:**
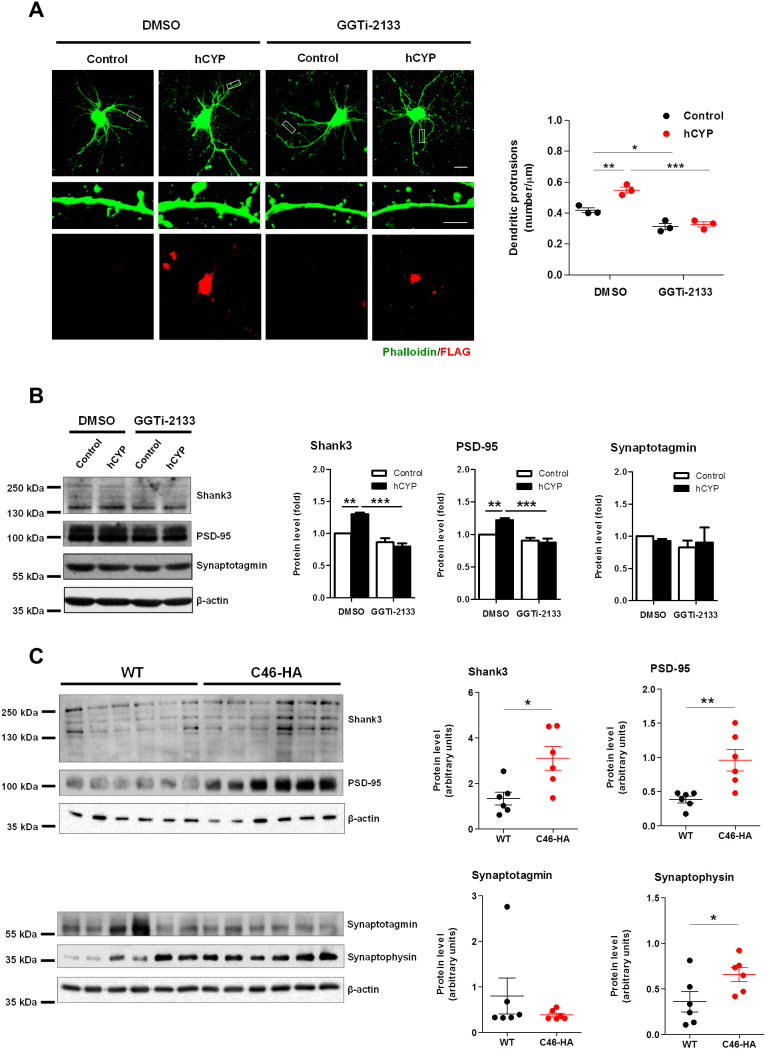
CYP46A1 increases the number of dendritic protrusions and induces synaptic proteins enrichment in a GGTase-I dependent manner. (**A,B**) Primary cultures of rat cortical neurons 19 days *in vitro* (19 DIV) were transfected with Control or hCYP vector and maintained for 48 hours. 24 hours after transfection cells were incubated with 100 nM GGTi-2133 or vehicle (DMSO). Statistical analysis was performed by two-way ANOVA followed by Tukey post-hoc test (*p < 0.05 **p < 0.01 ***p < 0.001). (**A**) Quantitation of dendritic protrusion density. Dendritic protrusions were quantified in 21 DIV neurons stained with DAPI and phalloidin (F-actin stain), and immunostained with anti-FLAG antibody. Images are representative of phalloidin staining (green) in Control and hCYP transfected neurons treated with DMSO or GGTi-2133. Scale bar: 20 μm (upper panel) and 5 μm (lower panel). Data represents mean values ± SEM from at least three independent experiments and is expressed as number of dendritic protrusions per μm of the respective dendrite. (**B**) Determination of synaptic protein levels in crude synaptosomal fractions (P2). P2 fractions isolated from 21 DIV neurons were subjected to Western Blot analysis for the postsynaptic proteins PSD-95 and Shank3, and the presynaptic protein Synaptotagmin. β-actin was used as loading control. Data represents mean values ± SEM from at least three independent experiments and is expressed as fold to the control. (**C**) Synaptic proteins content in P2 fractions of WT and C46-HA mice. P2 fractions of cortical tissue isolated from WT and C46-HA mouse brain were subjected to Western Blot analysis for the postsynaptic proteins PSD-95 and Shank3, and the presynaptic proteins Synaptophysin and Synaptotagmin. β-actin was used as loading control. Data represents mean values ± SEM from at least three independent experiments and is expressed as arbitrary units. Statistical analysis was performed by Student’s *t*-test (*p < 0.05 **p < 0.01).

Since we found that ectopic CYP46A1 expression in neurons leads to an increase in dendritic arbor complexity, we investigated if this would also affect dendritic protrusion density. Thus, mature neurons (19 DIV) were transfected with Control or hCYP plasmids, maintained for 48 hours, and in the last 24 hours after transfection, cells were incubated with 100 nM GGTi-2133 or DMSO. Quantification of the number of dendritic protrusions was done at 21 DIV after neurons were fixed and stained with DAPI, phalloidin (F-actin stain) and immunostained with anti-FLAG antibodies (Fig. 2A). Our results show that the number of dentritic protusions increased by ~30% in hCYP-transfected neurons and that inhibition of prenylation not only decreased dentritic protrusion density but also impaired the CYP46A1-dependent effect (two-way ANOVA *p* < 0.05, Tukey HSD post hoc test).

Dendritic protrusions constitute the primary loci of excitatory synaptic transmission in the mammalian central nervous system. We therefore analysed if the increase in dentritic protusion density induced by CYP46A1 overexpression was accompanied by an enrichment of synaptic proteins in the crude synaptosomal fraction (P2) isolated from these cells. The relative abundance of postsynaptic proteins PSD95 and Shank3, and of the presynaptic protein synaptotagmin was analyzed by Western blot using specific antibodies (Fig. 2B). We observed a small but consistent and significant enrichment of PSD-95 and Shank3 in the isolated neuronal P2 fraction. The levels of synaptotagmin did not change between Control and hCYP-transfected neurons. Once again, treatment with GGTi-2133 impaired the CYP46A1-dependent increase in PSD-95 and Shank3 levels (two-way ANOVA *p* < 0.01, Tukey HSD post hoc test). To further confirm our observations, we isolated the crude synaptosomal fraction from the brain cortex of WT and transgenic C46-HA mice, and determined by immunoblotting the content of post- and presynaptic proteins (Fig. 2C). In agreement with the *in vitro* data, we observed an increase in PSD-95 and Shank3 expression in C46-HA mouse brain relative to WT controls. Additionally, we also observed an increase in the presynaptic protein synaptophysin (Fig. 2C).

These results indicate that CYP46A1 promotes an increase in dendritic protrusion density, accompanied by an enrichment of synaptic proteins at the synaptosomal level, in a GGTase-I dependent fashion.

### CYP46A1 promotes phosphorylation of Trk and consequently increases GGTase-I activity

**Figure 3:**
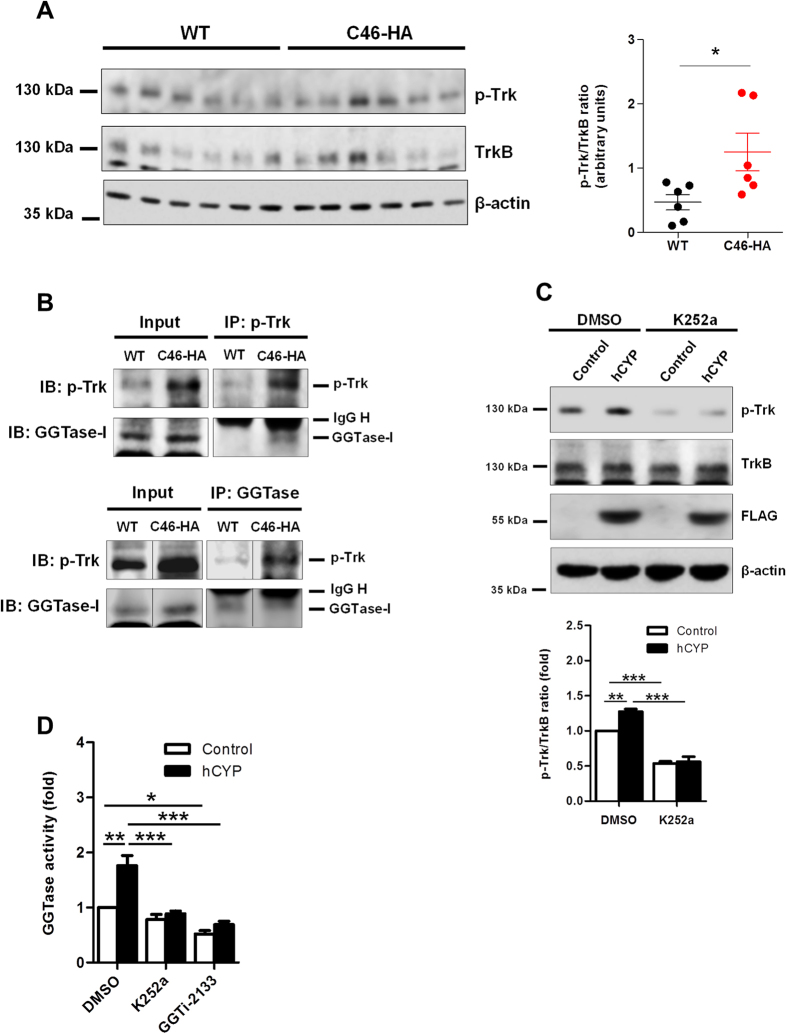
CYP46A1 induces phosphorylation of Trk *in vivo* and *in vitro* consequently increasing GGTase-I activity. (**A**) Total protein extract obtained from WT and C46-HA mice brain were subjected to Western Blot analysis for p-Trk and TrkB. β-actin was used as loading control. Data represents mean values ± SEM from at least three independent experiments and is expressed as fold of p-Trk/TrkB ratio relative to the control. Statistical analysis was performed by Student’s *t*-test (*p < 0.05). (**B**) Analysis of the interaction between p-Trk and GGTase-I proteins in the brains of WT and C46-HA mice. Immunoprecipitation (IP) experiments were carried out using total extracts from WT and C46-HA mouse brain and the anti-p-Trk and -GGTase-I antibodies (upper and lower panel, respectively). Resulting immunocomplexes were probed with p-TrkB or GGTase-I antibodies, and representative images of at least three independent experiments are shown. Input corresponds to 5% of tissue lysate and IgG H refers to the heavy chain of the antibodies used for IP. In the lower panel, the black lines (−) correspond to cropping lanes between samples that run in the same gel, under the same experimental conditions. (**C,D**) Primary cultures of rat cortical neurons 4 DIV were transfected with Control or hCYP vector and maintained for 48 hours. 24 hours after transfection, cells were incubated with 100 nM K252a, GGTi-2133, or vehicle (DMSO). Statistical analysis was performed by one- and two-way ANOVA followed by Tukey post-hoc test (*p < 0.05 **p < 0.01 ***p < 0.001). (**C**) Determination of p-Trk and TrkB levels in neuronal total protein extracts, by Western blot analysis. β-actin was used as loading control. Data represents mean values ± SEM from at least three independent experiments and is expressed as the p-Trk/TrkB ratio in arbitrary units. (**D**) Assessment of GGTase-I activity in neurons. Equal amounts of total extracts from 6 DIV neurons were subjected to a GGTase-I fluorometric assay. Data represents mean values ± SEM from at least three independent experiments and is expressed as fold to the control.

Cholesterol reduction was shown to increase phosphorylation and activation of Trk receptor in hippocampal neurons *in vitro*^17^. Additionally, BDNF-dependent activation of the TrkB receptor has been demonstrated to promote the physical interaction between TrkB and GGTase-I, and consequently, to increase GGTase-I prenylation activity^16^. Therefore, we hypothesized that in addition to enhancing sGTPase activity due to the increased isoprenoid pool^15^, CYP46A1 could also induce GGTase-I activity, as a result of Trk activation and consequent interaction with GGTase-I. Therefore, we started by assessing the phosphorylated Trk (p-Trk) levels in WT and C46-HA mouse brain, by Western blot analysis. We observed a significant increase in Trk phosphorylation levels in total protein extracts prepared from C46-HA mouse brain cortex (Fig. 3A). To test if the CYP46A1-dependent increase in p-Trk levels could affect protein-protein interaction between Trk and GGTase-I *in vivo*, we performed co-immunoprecipitation (Co-IP) assays (Fig. 3B). Our results show increased association between p-Trk and GGTase-I in C46-HA mouse brain. Indeed, immunoprecipitation (IP) of p-Trk caused the Co-IP of GGTase-I in total protein extracts of C46-HA mouse brain, while in the same experimental conditions, Co-IP of GGTase-I could not be detected in WT brain extracts (Fig. 3B). Additionally, IP of GGTase-I led to a Co-IP of p-Trk in both WT and C46-HA brain extracts, although in C46-HA the amount of p-Trk that co-immunoprecipitated with GGTase-I was higher compared to WT. The levels of p-Trk were increased in the total brain extracts of C46-HA mice (Fig. 3B), as previously observed (Fig. 3A), which led to the detection of higher levels of p-Trk after the IP of p-Trk itself or GGTase-I (Fig. 3B), reflecting an increased association of p-Trk with GGTase-I in the brains of C46-HA mice. Furthermore, to determine if the p-Trk-GGTase-I interaction induced by CYP46A1 positively affects GGTase-I activity, hCYP-transfected 6 DIV neurons were incubated with 100 nM K252a, a widely used inhibitor of Trk phosphorylation, for 24 hours, and the phosphorylation levels of Trk and GGTase-I activity were determined (Fig. 3C,D). Transfection of hCYP led to a modest but significant increase in Trk phosphorylation, in accordance with the *in vivo* data. However, when neurons were incubated with K252a, this effect could no longer be observed (Fig. 3C; two-way ANOVA *p* < 0.05, Tukey HSD post hoc test). Concomitantly, GGTase-I activity was increased ~2-fold in hCYP-transfected neurons, however, upon treatment with K252a, this effect was abolished. GGTi-2133 was used as a negative control for GGTase-I activity (Fig. 3D; one-way ANOVA *p* < 0.001, Tukey HSD post hoc test). Thus, CYP46A1 leads to an increase in GGTase-I activity, which is dependent on Trk phosphorylation and interaction between both proteins.

### Trk activation is required for CYP46A1-dependent increase on dendritic outgrowth and partially necessary for its effect on dendritic protrusions

**Figure 4:**
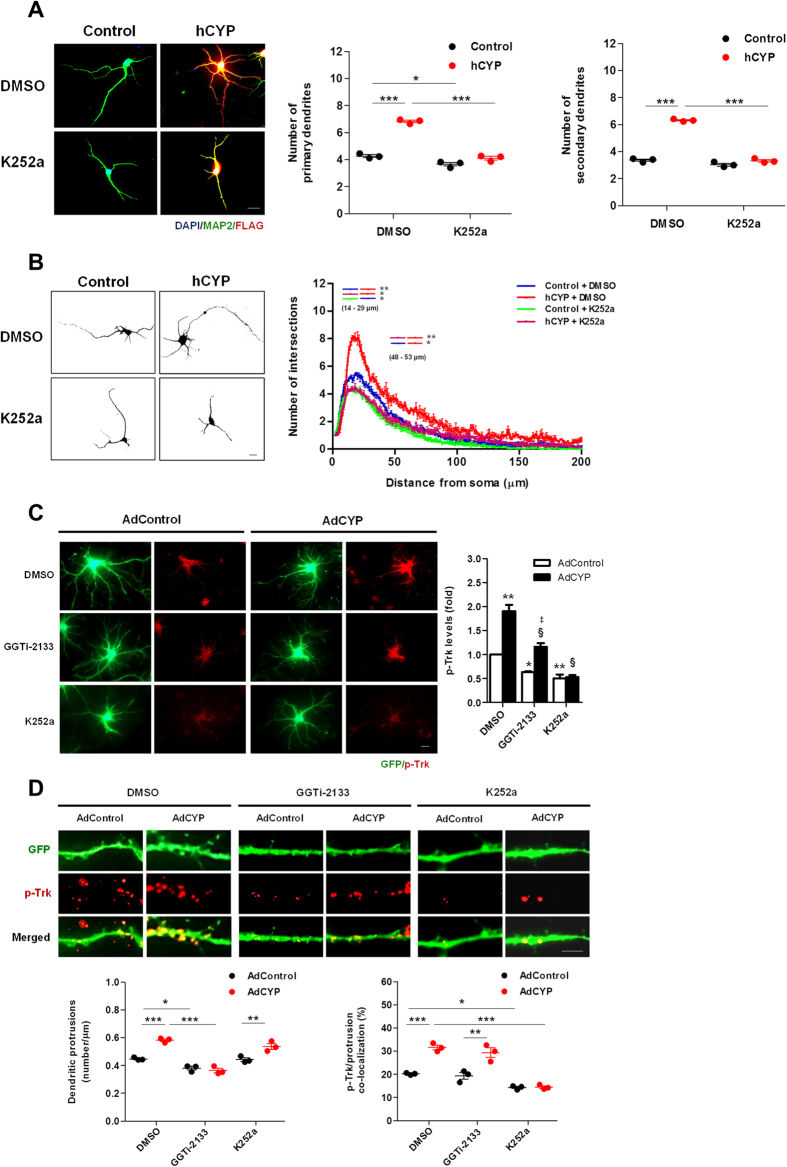
Trk activation is required for CYP46A1-dependent increase on dendritic outgrowth and partially necessary for its effect on dendritic protrusions. (**A,B**) Primary cultures of rat cortical neurons 4 DIV were transfected with Control or hCYP vector and maintained for 48 hours. 24 hours after transfection, cells were incubated with 100 nM of K252a or vehicle (DMSO). Neurons were stained with DAPI (blue) and immunostained with anti-FLAG (red) and -MAP2 (green) antibodies for dendritic outgrowth analysis at 6 DIV. Statistical analysis was performed by two-way ANOVA and post-hoc Tukey test (*p < 0.05 **p < 0.01 ***p < 0.001). Scale bar: 20 μm. (**A**) Quantitation of primary and secondary dendrites of analysed neurons. Representative merged images are shown. Data represents mean values ± SEM from at least three independent experiments and is expressed as number of dendrites. (**B**) Quantitation of dendritic arbor complexity by Sholl analysis. Images are representative of 6 DIV neurons and data represents mean values ± SEM from at least three independent experiments and is plotted as number of intersections versus distance (μm) to soma. (**C,D**) Primary cultures of rat cortical neurons 19 DIV were transduced with AdControl or AdCYP adenovirus and maintained for 48 hours. 24 hours after transduction cells were incubated with 100 nM GGTi-2133, K252a or vehicle (DMSO). Neurons were immunostained with anti-p-Trk (red). Statistical analysis was performed by one-way ANOVA followed by Tukey post-hoc test. (**C**) Quantitation of p-Trk levels. Images are representative of 21 DIV neurons exhibiting GFP (green). p-Trk levels were determined by measuring the fluorescence and normalizing by the area of the respective neuron. Scale bar: 20 μm. Data represents mean values ± SEM from at least three independent experiments and is expressed as fold change over control (*p < 0.05 **p < 0.01 vs Control-DMSO; ^§^*p* < 0.001 vs hCYP-DMSO; ^‡^*p < *0.01 vs Control-GGTi-2133). (**D**) Quantitation of dendritic protrusion density of 21 DIV neurons exhibiting GFP. The percentage of co-localization between protrusions and p-Trk immunostaining was determined. Scale bar: 5 μm. Representative images are shown and data represents mean values ± SEM from at least three independent experiments and is expressed as number of protrusions per μm of the respective dendrite (left chart) and percentage of co-localization between p-Trk and protrusions (right chart) (*p < 0.05 **p < 0.01 ***p < 0.001).

To determine how crucial Trk activation is for CYP46A1-dependent increase on dendritic outgrowth and protrusion density in neurons, we transfected 4 DIV neurons with Control or hCYP vectors. Cells were maintained for 48 hours, and incubated with 100 nM K252a or vehicle in the last 24 hours after transfection (Fig. 4A,B). As expected, the number of primary and secondary dendrites was higher in neurons transfected with hCYP, and this effect was abolished when Trk phosphorylation was inhibited by K252a treatment (Fig. 4A; two-way ANOVA *p* < 0.001, Tukey HSD post hoc test). Moreover, Sholl analysis revealed that the increase in arbor complexity induced by CYP46A1 was inhibited in the presence of K252a (Fig. 4B; two-way ANOVA *p* < 0.05, Tukey HSD post hoc test). These results, together with data in Fig. 1, indicate that both Trk and GGTase-I activity are essential for the CYP46A1 effect on dendritic outgrowth. This is consistent with the fact that CYP46A1 led to an increased interaction between the two proteins, and to the activation of GGTase-I. Furthermore, we also investigated if Trk inhibition could impair CYP46A1-dependent increase on dendritic protrusion density (Fig. 4C,D). To facilitate the analysis and increase the number of 19 DIV neurons expressing CYP46A1, we generated a recombinant adenovirus to express FLAG-tagged CYP46A1. Mature neurons were transduced with an adenovirus encoding GFP (AdControl), or encoding GFP and FLAG-tagged CYP46A1 (AdCYP) (expressed from two independent promoters) and maintained for 48 hours. The levels of CYP46A1 expression were increased ~3 fold, paralled by a significant increase in the levels of 24OHC in culture media (Supplementary Fig. 3). In the last 24 hours, neurons were incubated with 100 nM GGTi-2133 or K252a and then fixed and immunostained for p-Trk. In agreement with our previous data from transient transfected 4 DIV neurons, p-Trk was increased in 19 DIV neurons transduced with AdCYP (Fig. 4C). Interestingly, in the presence of GGTi-2133, this effect was maintained, although, the overall p-Trk levels were decreased. As expected, when cells were incubated with K252a, p-Trk levels were strikingly decreased and the CYP46A1-dependent increase on Trk phosphorylation was abolished (one-way ANOVA *p* < 0.001, Tukey HSD post hoc test). These results are consistent with the fact that GGTase-I activation is a downstream consequence of Trk phosphorylation induced by CYP46A1, and inhibition of GGTase-I *per se* does not influence CYP46A1 effect on p-Trk. Additionally, we also confirmed that the synaptic proteins PSD-95 and Shank3 are also increased in the P2 fraction of these AdCYP neurons (Supplementary Fig. 4), as previously assessed for hCYP-transfected 21 DIV neurons. For the dendritic protrusion density analysis, we took advantage of transduced neurons expressing GFP, which allowed us to determine the number of protrusions and assess their co-localization with p-Trk in the different conditions tested (Fig. 4D). As previously shown, GGTi-2133 decreased the density of protrusions and abolished the increment induced by CYP46A1, while K252a has no effect (one-way ANOVA *p* < 0.001, Tukey HSD post hoc test). Furthermore, CYP46A1 strikingly increased p-Trk at dendritic protrusions, as observed by the percentage of co-localization of these structures with p-Trk. Interestingly, although GGTi-2133 decreased the number of protrusions, it did not alter the percentage of co-localization between protrusions and p-Trk. On the other hand, K252a reduced the percentage of protrusions that co-localize with p-Trk, but not the number of these structures (two-way ANOVA *p* < 0.001, Tukey HSD post hoc test). Thus, the increase in the number of dendritic protrusions elicited by CYP46A1 does not seem to dependent on Trk activation, and consequently, on the activation of the Trk-GGTase-I axis. Since we have already described that CYP46A1 activates the mevalonate pathway as a consequence of membrane cholesterol reduction, inducing prenylation and activation of sGTPases^15^, it is plausible that this mechanism might be sufficient on itself to drive a sGTPases-mediated remodeling of spines, occurring independently of Trk-induced GGTase-I activation.

In summary, CYP46A1 increases the number of dendritic protrusions and the presence of p-Trk in these structures. Inhibition of either Trk or GGTase-I does not completely abolish CYP46A1 effect, but partially affects it in a specific manner.

### CYP46A1-induced dendritic outgrowth is triggered by the reduction in cellular cholesterol levels

**Figure 5:**
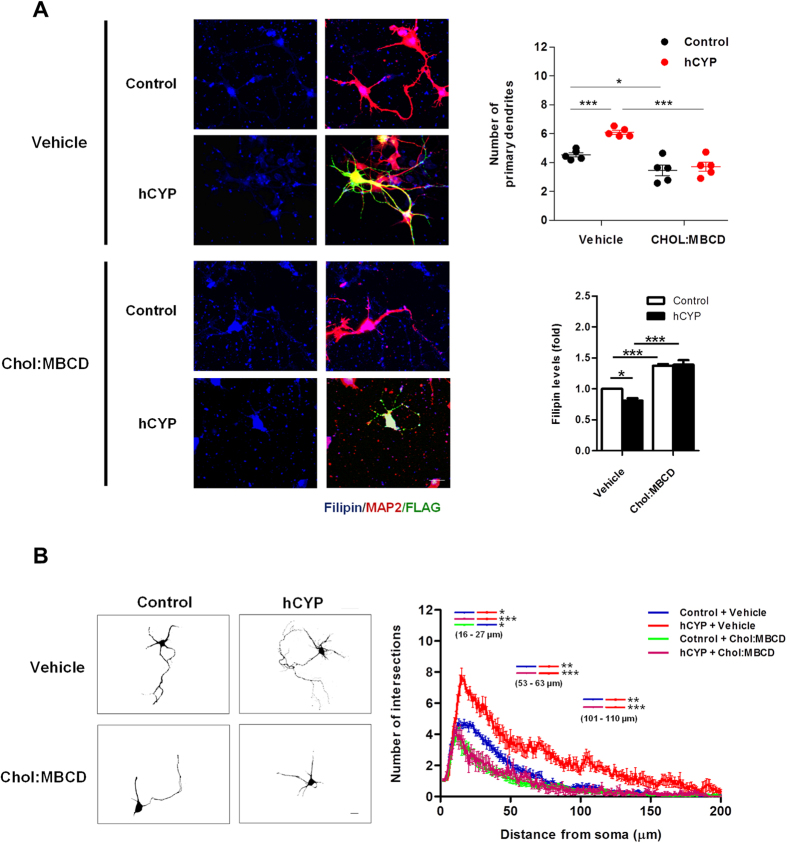
Cholesterol reduction is necessary for CYP46A1 induced dendritic outgrowth. (**A,B**) Primary cultures of rat cortical 4DIV neurons were transfected with Control or hCYP vector and maintained for 48 hours. 24 hours after transfection cells were incubated with 10 μM cholesterol (Chol:MBCD). Neurons were stained with filipin III (free cholesterol staining) and immunostained for MAP2 and FLAG. Scale bar: 20 μm. Statistical analysis was performed by two-way ANOVA followed by Tukey post-hoc test (*p < 0.05 **p < 0.01 ***p < 0.001) (**A**) Quantitation of filipin III levels and primary dendrites. Representative merged images of filipin III (blue), FLAG (green) and MAP2 (red) staining, in Control and hCYP transfected 6DIV neurons supplemented with vehicle or 10 μM Chol:MBCD. Average filipin III fluorescence intensity was measured in neurons and normalized to the respective area. Data represents mean values ± SEM from at least three independent experiments and is expressed as fold change over control. (**B**) Quantitation of dendritic arbor complexity. Sholl analysis was performed in neurons transfected with Control or hCYP vector and treated with vehicle or Chol:MBCD. Images are representative of 6 DIV neurons in each condition. Data represents mean values ± SEM from at least three independent experiments and is plotted as number of intersections versus distance (μm) to soma.

To confirm if the trigger for the overall effect of CYP46A1 is dependent on the reduction of cholesterol levels, 4 DIV neurons transfected with hCYP and maintained for 48 hours, were cultured for the last 24 h in media supplemented with 10 μM cholesterol (Chol:MBCD). Neurons were fixed and stained with filipin III (used to localize free cholesterol) and immunostained with anti-MAP2 antibody to analyze dendritic outgrowth (Fig. 5). We have previously reported that ectopic expression of CYP46A1 reduces cholesterol in the membrane fraction of neuronal cells^15^. Herein, although we could not detect differences in the level of free cholesterol in total extracts from cultures containing hCYP-transfected neurons (Supplementary Fig. 5), by analyzing filipin III staining in neurons that were specifically positive for FLAG staining, ie, transfected with hCYP, we could observed a 20% reduction in cholesterol levels (Fig. 5A). This decrease is paralleled by an increment in the number of primary dendrites (Fig. 5A) and a higher dendritic arbor complexity, determined by Sholl analysis (Fig. 5B). The induction of dendritic outgrowth by CYP46A1 is abolished upon cholesterol supplementation to neurons. Moreover, higher levels of neuronal cholesterol, due to media supplementation, have a negative effect on dendritic outgrowth. (Fig. 5A,B) (two-way ANOVA p < 0.05, Tukey HSD post hoc test). Although cholesterol accumulation might render a toxic effect or an increased susceptibility to toxic stimuli^11^,^18^,^19^,^20^, besides the morphological differences, we did not detect significant changes in cell death markers, namely lactate dehydrogenase (LDH) or adenylate kinase (AK) activity in culture media, between cholesterol supplementation conditions and respective controls (Supplementary Fig. 6). These data indicate that cholesterol reduction is the necessary trigger for the increase of dendritic outgrowth mediated by CYP46A1.

### The effect of CYP46A1 on dendritic protrusions and p-Trk levels is dependent on cholesterol reduction

**Figure 6:**
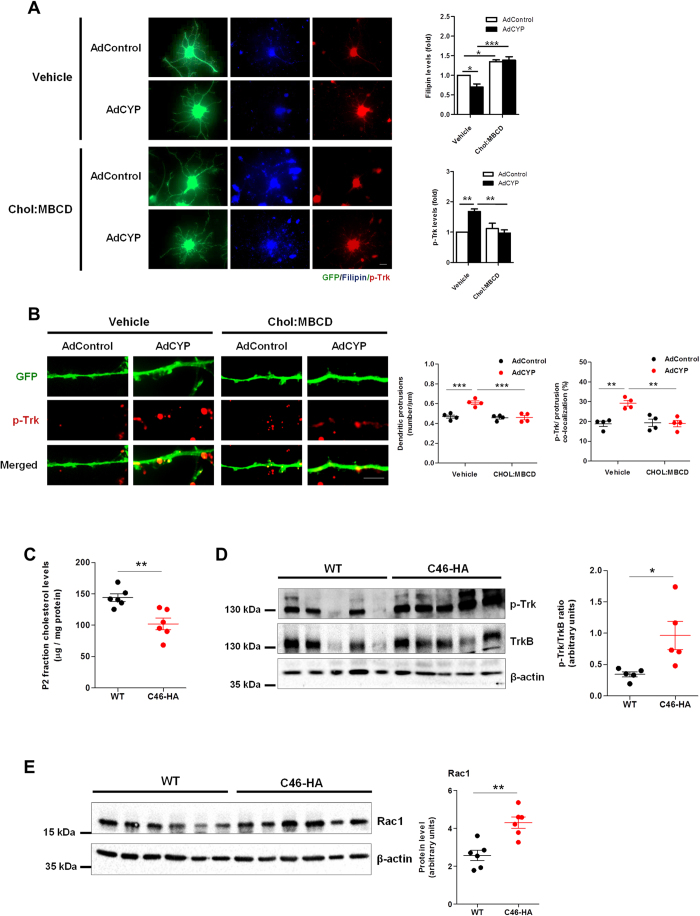
Cholesterol reduction is a necessary trigger for the CYP46A1-dependent increase in dendritic protrusion density and p-Trk levels. (**A,B**) Primary cultures of rat cortical neurons 19 DIV were transduced with AdControl or AdCYP adenovirus and maintained for 48 hours. 24 hours after transduction cells were incubated with 10 μM Chol:MBCD. Neurons were stained with filipin III (blue) and immunstained with p-Trk (red). Statistical analysis was performed by two-way ANOVA followed by Tukey post-hoc test (*p < 0.05 **p < 0.01 ***p < 0.001). (**A**) Quantitation of filipin III and p-Trk levels. Images are representative of 21 DIV neurons exhibiting GFP (green), stained with filipin III and immunostained with p-Trk. Filipin III and p-Trk levels were determined by measuring the average fluorescence intensity in neurons and normalized to the respective area. Scale bar: 20 μm. Data represents mean values ± SEM from at least three independent experiments and is expressed as fold change over control. (**B**) Quantitation of dendritic protrusion density. Dendritic protrusions of 21 DIV neurons exhibiting GFP and immunostained for p-Trk were quantified and the percentage of co-localization with p-Trk immunostaining was determined. Images are representative of GFP and p-Trk immunostaining in AdControl and AdCYP-transduced neurons treated with vehicle or cholesterol. Scale bar: 5 μm. Data represents mean values ± SEM from at least three independent experiments and is expressed as number of protrusions per μm of the respective dendrite. (**C**) Cholesterol content in crude synaptosomal (P2) fraction of WT and C46-HA mouse brain. Total cholesterol levels were determined with the Amplex^®^Red cholesterol determination kit in P2 fractions obtained from brain cortex of WT and C46-HA mice. Data represents mean values ± SEM and is expressed as μg of cholesterol per mg of protein. Statistical analysis was performed by Student’s *t*-test (**p < 0.01). (**D,E**) Crude synaptosomal (P2) fractions of WT and C46-HA mouse brain were subjected to Western Blot analysis for p-Trk and TrkB (**D**) and Rac1 (**E**). Data represents mean values ± SEM and is expressed as p-Trk/TrkB ratio or Rac1 levels in arbitrary units. β-actin was used as loading control. Statistical analysis was performed by Student’s *t*-test (*p < 0.05 **p < 0.01).

Since we found that a reduction in cholesterol levels is essential for the CYP46A1-mediated increase of dendritic outgrowth, we wanted to confirm if the trigger for the overall effect of CYP46A1 on dendritic protrusions and p-Trk was also dependent on the reduction of cholesterol levels. 19 DIV neurons transduced with AdCYP and maintained for 48 hours, were supplemented with 10 μM cholesterol (Chol:MBCD) in the culture media during the last 24 hours. Neurons were fixed and stained with filipin III and immunostained with an anti-p-Trk antibody (Fig. 6A,B). Similarly to what was observed in 4 DIV neuronal cultures, cholesterol levels in total extracts obtained from cultures transduced with AdCYP do not change when compared to the control (Supplementary Fig. 7). However, when analyzing neurons specifically transduced with AdCYP using filipin III, we observed a decrease in free cholesterol levels (Fig. 6A). Consistent with previous data^17^, this decrease in cholesterol is concomitant with increased p-Trk levels. When the culture media was supplemented with cholesterol, we could no longer observe the decrease in filipin staining and increased Trk phosphorylation induced by CYP46A1 (two-way ANOVA *p* < 0.05, Tukey HSD post hoc test). Furthermore, cholesterol supplementation also abolished the increase in dendritic protrusion density and p-Trk levels in these structures triggered by CYP46A1 (Fig. 6B, two-way ANOVA *p* < 0.01, Tukey HSD post hoc test). This was also confirmed *in vivo* (Fig. 6C,D). Firstly, we found a reduction of cholesterol levels in the crude synaptossomal P2 fraction isolated from C46-HA mouse brain cortex when comparing to the WT (Fig. 6C). Strikingly, we detected a 3-fold increase in the levels of phosphorylated Trk in the P2 fraction (Fig. 6D). Interestingly, levels of Rac1, a Rho GTPase well known for its role in neuronal function and morphological events, including dendritic outgrowth and spine formation^21^,^22^,^23^,^24^, were also significantly increased in the P2 fraction of C46-HA mouse brain cortex (Fig. 6E). This is in line with the fact that CYP46A1 induces significant morphological changes, namely the increase in dendritic protrusion density.

These data indicate that the reduction in cellular cholesterol mediated by CYP46A1 is the crucial trigger for the subsequent increase in p-Trk levels and protrusion density.

### The effect of neuronal cholesterol metabolism mediated by CYP46A1 on the expression levels of synaptic genes

**Figure 7:**
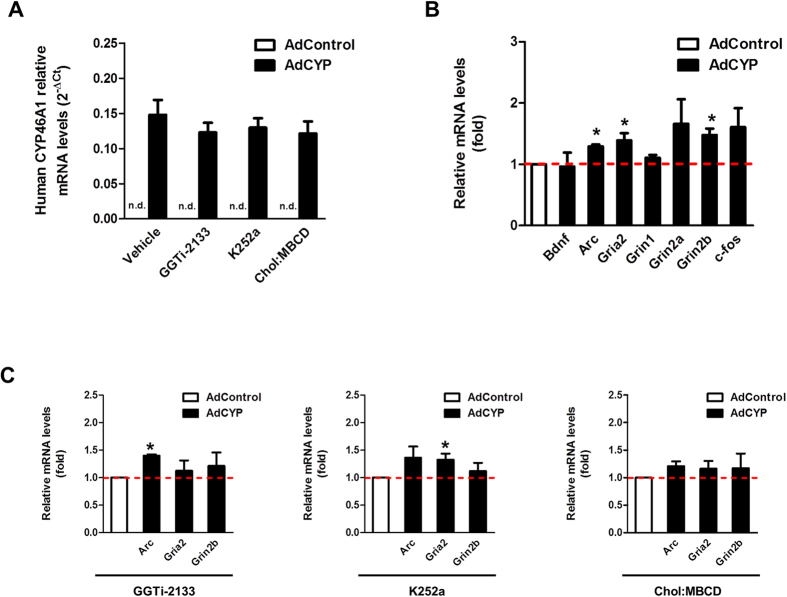
CYP46A1 increases synaptic genes mRNA levels. Real-time PCR (qPCR) analysis was performed in primary cultures of rat cortical neurons 19 DIV transduced with AdControl or AdCYP adenovirus and maintained for 48 hours. (**A**) qPCR analysis of human *CYP46A1* mRNA levels. Data represents mean values ± SEM from at least three independent experiments and is expressed as 2^−ΔCt^. (**B**) qPCR analysis of Bdnf, Arc, Gria2, Grin1, Grin2a, Grin2b and C-fos mRNA levels. Values were normalized to the internal standard GAPDH Data represents mean values ± SEM from at least three independent experiments and is expressed as fold to the AdControl. Statistical analysis was performed by Student’s *t*-test (*p < 0.05). (**C**) qPCR analysis of Arc, Gria2 and Grin2ba mRNA levels. 24 hours after transduction cells were incubated with 100 nM GGTI-2133, 100 nM K252a or 10 μM Chol:MBCD. Values were normalized to the internal standard GAPDH. Data represents mean values ± SEM from at least three independent experiments and is expressed as fold to the AdControl. Statistical analysis was performed by Student’s *t*-test (*p < 0.05).

Since we found that CYP46A1 increases synaptic proteins and dendritic protrusion density, we decided to determine the mRNA profile of genes associated with synaptic function in 19 DIV neurons transduced with AdCYP and maintained for 48 hours. We confirmed the ectopic expression of the human CYP46A1 (Fig. 7A), and observed comcomitant significant changes in the mRNA levels of Arc, Gria2 and Grin2b of ~1.3, 1.4 and 1.5 fold, respectively (Fig. 7B). Since the previously described neuronal effects induced by CYP46A1 rely primarly on cholesterol reduction, and subsequently on Trk activation and/or GGTase-I activity, we analyzed if the alterations on gene expression levels induced by CYP46A1 were altered in the presence GGTi-2133, K252a, or cholesterol supplementation. Importantly, we observed that none of these compounds affect the basal mRNA levels of Arc, Gria2 or Grin2b (Supplementary Fig. 8). Interestingly, neurons transduced with AdCYP and incubated with 100 nM GGTi-2133 did not exhibit increments in Gria2 and Grin2b mRNA levels, although expression levels of Arc were increased by ~1.4 fold (Fig. 7C). On the other hand, in the presence of 100 nM K252a, neurons transduced with AdCYP revealed an increase in Gria2 mRNA levels by ~1.3 fold, while Arc and Grin2b levels did not change (Fig. 7C). Importantly, AdCYP transduced neurons incubated with media supplemented with 10 μM cholesterol (Chol:MBCD), did not exhibit any increase in the mRNA levels of Arc, Gria2 and Grin2b (Fig. 7C). Taken together, these results indicate that cholesterol reduction is necessary for the overall changes in gene expression elicited by CYP46A1, although the specific underlying mechanism seem to differ between different genes.

All these data from 6 and 22 DIV neurons transfected and transduced, respectively, with CYP46A1, and incubated with cholesterol-supplemented media, suggest that cholesterol reduction is the primary and crucial trigger for the neuronal effects arising from CYP46A1 expression concerning dendritic outgrowth and synaptic function.

## Discussion

CYP46A1 is the major regulator of brain cholesterol elimination. Although the underlying mechanisms are not clear, increased levels of this enzyme have been extensively associated with improved cognitive function in physiological and pathological conditions^10^,^13^,^20^. For this reason, CYP46A1 has been envisioned as an appealing drug target for the treatment of neurodegenerative disorders.

This study uncovers the molecular basis underlying the impact of CYP46A1 on high-order brain functions. Indeed, herein we describe that an increase in CYP46A1 expression induces neuronal dendritic outgrowth, dendritic protrusion density, synaptic genes, and elicits an increase of synaptic proteins in the synaptosomal fraction *in vivo* and *in vitro*. All of these morphological and molecular changes promoted by increased CYP46A1 expression are in line with the enhancement in cognitive function observed *in vivo* in CYP46A1 transgenic mice^13^ and with the cognitive deficit observed in Cyp46a1^−/−^ mice^12^. Strikingly, all changes in neuronal morphology and synaptic proteins induced by CYP46A1, are impaired by the pharmacological inhibition of GGTase-I activity. This is coherent with the fact that CYP46A1 increases geranylgeranylation of sGTPases by activating the mevalonate pathway, as previously described by our group^15^. Moreover, a recent report shows that administration of GGTi-2147, a GGTase-I inhibitor, inhibits basal- and activity-dependent dendritic spine formation *in vivo* and, more importantly, decreases learning and memory^25^, highlighting an important role of this prenyltransferase in the CNS.

Analysis of brain cortex tissue from C46-HA mice, and primary cultures of rat cortical neurons transfected or transduced with vectors encoding CYP46A1, led us to the conclusion that CYP46A1 induces Trk phosphorylation. It is conceivable that the mild decrease in membrane cholesterol content induced by CYP46A1 affects membrane lipid composition. In turn, this might promote higher chances of Trk clustering and therefore favor dimerization, facilitating its auto-phosphorylation. It has been suggested that the increase in membrane sphyngomyelin:cholesterol ratio induces TrkB activation in neurons^26^. The authors propose that, since sphyngomyelin can facilitate lipid packaging, it might favour TrkB activation, and demonstrated that increased levels of sphyngomyelin is an important mechanism for sustaining TrkB activity, in addition to BDNF activation. In line with this idea, CYP46A1-induced reduction of membrane cholesterol might increase the ratio sphingomylein:cholesterol ratio and lead to a ligand-free Trk activation. These receptors regulate several features in the CNS, such as synaptic strength and plasticity or neuronal survival and differentiation^27^,^28^. Furthermore, it has been reported that BDNF-dependent TrkB activation leads to an interaction with GGTase-I, increasing its prenylation activity, which was shown to be essential for dendritic outgrowth^16^. These reports prompted us to determine if Trk phosphorylation induced by CYP46A1 was also translated into higher interaction with GGTase-I, and to increased geranylgeranylation activity. Indeed, Co-IP experiments using extracts from the brain cortex of C46-HA mice show that there is an increased protein association between p-Trk and GGTase-I, probably due to the overall increase in p-Trk levels in the brains of C46-HA mice. Accordingly, ectopic expression of CYP46A1 *in vitro* increases GGTase-I activity, and this effect is abolished by the pharmacological inhibition of Trk receptor. Importantly, the phosphorylation of Trk mediated by CYP46A1 is not affected by GGTase-I inhibition, which confirms a causal relationship. Therefore, activation of the p-Trk-GGTase-I axis by CYP46A1, implies firstly the phosphorylation of Trk, which consequently leads to increased GGTase-I activity. One of the aims of this work was to evaluate the changes in dendritic outgrowth in response to an acute increase of CYP46A1 expression, however, it would also be interesting, in the future, to characterize the dendritic arbor throughout neuronal maturation, in neurons subjected to a sustained increase of CYP46A1 levels.

Although the p-Trk-GGTase-I axis is shown to be crucial for increased dendritic outgrowth mediated by CYP46A1, the increase in dendritic protrusion density elicited by CYP46A1 is not dependent on Trk phosphorylation. On the other hand, inhibition of GGTase-I abolishes the increased protrusion density mediated by CYP46A1, but maintains the increase in the percentage of p-Trk that co-localizes with dendritic protrusions. Morphogenesis and maintenance of these dynamic structures is intimately linked to the degree of actin polymerization, which in turn is tightly regulated by sGTPases of the Rho-family^21^,^29^. Therefore, it is plausible that the activation of the mevalonate pathway and subsequent activation of sGTPases induced by CYP46A1^15^ is sufficient for the specific morphological effect of increasing the number of protrusions, in a Trk-independent manner. Accordingly, we found a significant increase in Rac1 levels in crude synaptosomal fractions prepared from C46-HA mouse cortex.

Furthermore, consistent with the increase in synaptic proteins and dendritic protrusions we also observed that CYP46A1 increases the mRNA levels of the synaptic genes Arc, Gria2 and Grin2b, which is depedent on the reduction of cholesterol levels.

Indeed, cholesterol supplementation abolishes the increased mRNA levels of synaptic genes, Trk phosphorylation levels, the increase of dendritic outgrowth elicited by the p-Trk-GGTase-I axis and the induction of dendritic protrusion density mediated by GGTase-I. Moreover, we found a striking increase of p-Trk in the crude synaptosomal fraction prepared from brain cortex obtained from C46-HA mice. Although total cholesterol levels in the brains of C46-HA mice do not change^13^, cholesterol content in the synaptosomal P2 fraction is lower when compared to wild-type animals. These results, suggest that although CYP46A1 activity does not affect total cholesterol levels in the brain, it might be inducing permanent changes at particular cells and/or sub-cellular compartments.

Furthermore, since it has been shown that 24OHC has several effects on neuronal cells^15^,^30^,^31^,^32^,^33^, one could consider that CYP46A1-mediated effects might be underlined by an increased production of this oxysterol. However, we have found that 24OHC has the opposite effect of CYP46A1 regarding dendritic outgrowth *in vitro*, which further corroborates the idea that cholesterol loss *per se* is the trigger for the observed effects induced by CYP46A1.

It is widely known that cholesterol is essential for proper neuronal function. In fact, several postsynaptic processes important for synaptic function and morphology depend on cholesterol-rich lipid rafts^34^. Cholesterol reduction using 3-hydroxy-3-methylglutaryl co-enzyme A reductase inhibitors–statins-has led to conflicting data. Atorvastatin was reported to cause neurite loss^35^, and to induce neurite outgrowth in rat cortical neurons^36^ through different mechanisms. It was also reported that cholesterol depletion by MBCD increases neurite outgrowth in hippocampal neurons, while both cholesterol loading and depletion inhibits neurite outgrowth in cortical neurons^37^. Importantly, the statins/MBCD approach induces an artificial, abrupt and drastic cholesterol depletion that is not accompanied by a feedback activation of the mevalonate pathway as it happens by activating the endogenous pathway of brain cholesterol metabolism through increased CYP46A1 expression^13^,^15^,^38^. Regarding synaptic function, cholesterol reduction mediated by statins or acute extraction by MBCD causes an immediate collapse of spines^39^. However, it has been recently shown that the induction of LTP, mediated by the glutamate receptor N-methyl-D-aspartate receptor (NMDAR), leads to a 40–50% reduction in neuronal cholesterol which, in turn, leads to further synaptic potentiation. On the other hand, cholesterol supplementation completely abolishes glutamate-induced LTP^40^. Furthermore, a 20–30% reduction in cholesterol content through pharmacological manipulation, has been shown not to have any toxic effect on hippocampal neurons, in fact, it actually enhances excitatory synaptic transmission and facilitates LTP^40^. Interestingly, it has been established that cholesterol loss arising from glutamatergic synaptic input, is due to an increase in CYP46A1 activity^41^. These results are in line with our observations indicating that CYP46A1-mediated cholesterol loss increases the levels of synaptic proteins and genes, accompanied by an increment in dendritic protrusion density. Actually, cholesterol accumulation is deleterious to neurons. Increased membrane cholesterol of cultured neurons recapitulates Alzheimer’s early phenotype^18^ and increases neuronal susceptibility to beta-amyloid peptide-induced toxicity^19^. Furthermore, *in vivo* accumulation of cholesterol due to silencing of CYP46A1, leads to amyloidogenesis, neuronal death and cognitive impairment^11^. According to our results, CYP46A1-mediated cholesterol metabolism is beneficial for neuronal development and activity, which is in line with other *in vivo* studies^13^,^14^.

In conclusion, this study shows that brain cholesterol metabolism, orchestrated by CYP46A1, triggers an increase in neuronal dendritic outgrowth and synaptic markers, in a GGTase-I- and Trk-dependent fashion. Furthermore, we conclude that the overall effect of CYP46A1 in neurons arises from GGTase-I and Trk acting in both a concerted and independent fashion. These molecular events might underline major outcomes *in vivo*, such as changes in high-order brain functions. In fact, as also described in a previous report^17^, we have detected an increase in endogenous CYP46A1 expression during neuronal maturation *in vitro* which might underline, to some extent, morphological and functional changes that occur during this process *in vivo*, by activating sGTPases and Trk pathways. The understanding of how cholesterol homeostasis affects brain function is extremely relevant, particularly since many neurodegenerative and neurological disorders have brain cholesterol homeostasis dysregulation as a common feature.

## Materials and Methods

### Reagents and antibodies

The following primary antibodies were used: anti-FLAG^®^M2 (clone M2, F1804; Sigma-Aldrich Inc., St Louis, MO, USA), anti-microtubule-associated protein 2 (MAP2) (AB5622, Merck Millipore Corporation., Darmstadt, Germany), anti-postsynaptic density protein 95 (PSD95) (MAB1598, Merck Millipore), anti-SH3 and multiple ankyrin repeat domains 3 (Shank3) (sc-30193, Santa Cruz Biotechnology Inc., Santa Cruz, CA, USA), anti-Synaptotagmin (ab77314, Abcam, Cambridge, UK) anti-Synaptophysin (MAB5258, Merck Millipore), anti p-Trk (sc-8058; Santa Cruz), anti-GGTase-Iβ (sc-1899, Santa Cruz), anti-TrkB (sc-12, Santa Cruz) and anti-β-actin (A5541, Sigma). Geranylgeranyl pyrophosphate (GGPP) ammonium salt, Triton™ X-100, Tween^®^ 20, IGEPAL^®^, dimethyl sulfoxide (DMSO), filipin III, 24S-hydroxycholesterol and the chemical inhibitors GGTi-2133 and K252a were all purchased from Sigma. The phosphatase inhibitor cocktail (04906837001) and the protease inhibitor cocktails 1 and 2 (11697498001 and 11836170001, respectively) were purchased from Roche Diagnostics (GmbH, Penzberg, Germany). The fluorogenic substrate used for GGTase-I activity assay was dansyl-GCVLL (Biosynthesis, Lewisville, TX, USA). Phalloidin was obtained from Molecular Probes^®^, Thermo Fisher Scientific Inc. (Waltham, MA, USA).

### Cholesterol:Methyl-β-Cyclodextrin complexes

Cholesterol:methyl-β-cyclodextrin complexes (Chol:MBCD) were produced as previously described^42^. Briefly, 1 g methyl-β-cyclodextrin was dissolved in phosphate-buffered saline (PBS) and heated to 80 °C in water bath with continuous stirring. In parallel, 30 mg of cholesterol were dissolved in 400 μl isopropanol/chloroform (2:1). The cholesterol solution was added in aliquots of 50 μl to the MBCD solution, stirring until it solubilized completely before additional aliquots were added. After cooling, the final solution was stored at room temperature.

### Primary cultures of cortical neurons

Primary cultures of rat cortical neurons were prepared from 17- to 18-day-old Wistar rat fetuses as described previously^43^ with minor modifications. In short, pregnant rats were sacrificed in a CO_2_ chamber and the fetuses were collected in Hank’s balanced salt solution (HBSS) with Ca^2+^ and Mg^2+^ (Gibco™, Thermo Fisher) and rapidly decapitated. After removal of meninges and white matter, the brain cortex was collected in HBSS without Ca^2+^ and Mg^2+^ (HBSS-2). The cortex was then mechanically fragmented, transferred into a 0.05% trypsin solution, and incubated for 15 min at 37 °C. Following trypsinization, cells were washed twice in HBSS-2 containing 10% fetal bovine serum (FBS) and resuspended in Neurobasal medium, supplemented with 0.5 mM L-glutamine, 25 μM L-glutamic acid, 2% B-27 supplement, and 12 mg/mL gentamicin. Isolated neurons were plated with a density around 640/mm^2^ on culture plates pre-coated with poly-D-lysine, and maintained at 37 °C in a humidified atmosphere of 5% CO_2_. Half of the medium of the neuronal primary cultures was changed every 3–4 days. Glutamic acid was only added into the medium when plating the cells. All the subsequent media changes and cell treatments were free from glutamic acid. HBSS buffers, Neurobasal, B-27, L-glutamine, FBS and gentamicin were all purchased from Gibco™, Thermo Fisher Scientific Inc. (Waltham, MA, USA). L-glutamic acid and poly-D-lysine were purchased from Merck Millipore and Sigma, respectively.

### Transfection and transduction

Neurons were transfected with a control plasmid harboring a CMV promoter (pCMV) or the same plasmid encoding the FLAG-tagged hCYP46A1 (pCMV-FLAG-CYP46A1) (described in our previous work^15^) using Lipofectamine^®^ 2000 (Invitrogen, Thermo Fisher) according to manufacturer’s instructions. Briefly, culture media was changed to fresh neurobasal media with all the supplements previously mentioned, except for gentamicin. Subsequently, Opti-MEM^®^ (Gibco™) containing the mixture of Lipofectamine^®^ 2000 and DNA (2.5 μl: 1 μg) was added and cells were incubated for 4 hours at 37 °C in a humidified atmosphere of 5% CO_2_. Afterwards, the transfection media was discarded and regular neuronal media was added.

Adenovirus encoding GFP or both GFP and FLAG-tagged CYP46A1 were generated by using the pAdTrack/pAdEasy system. GFP and FLAG-tagged CYP46A1 are expressed from two independent promoters. Adenovirus titration was done by flow cytometry using Guava EasyCyte 5HT^®^ (Merck Millipore)^44^,^45^. Neurons were transduced overnight with adenovirus at a titer of 7.5 × 10^7^ green fluorescent units (gfu) per ml of media.

### Immunocytochemistry and image analysis

Neurons were fixed with a solution of 4% paraformaldehyde in PBS. Subsequently, cells were rinsed with PBS, and permeabilized with a solution of triton X-100 0.1% in PBS for 20 min. Afterwards, cells were rinsed with PBS once more and blocking solution (FBS 10% and Tween^®^ 20 0.05% in PBS) was added for 1 hour at room temperature. After blocking, the cells were incubated overnight at 4 °C with the respective primary antibodies diluted in blocking solution. Unbound primary antibodies were washed with PBS, and neurons were incubated with the respective secondary antibodies and dyes (DAPI for nuclear staining, filipin III for free cholesterol staining, or phalloidin for F-actin staining) diluted in blocking solution for 1 hour at room temperature. Fluorescence visualization was performed in an AxioScope.A1 microscope (Zeiss, Germany) with an AxioCam HRm camera (Zeiss). Morphological analysis, fluorescence and co-localization quantification were performed using NIH ImageJ 1.46r software. Dendritic protrusions number (either mushroom-shape, spines or filopodia) was counted manually using ImageJ in order to determine the protrusion density, corresponding to the number of protrusions per length unit of the respective dendrite.

Sholl analysis was performed using the plug-in “sholl analysis” for ImageJ software. The software determined the number of intersections between the dendritic arborization and concentric circles created around the neuronal soma. The increment distance between each circle was set to 1 μm. The values of intersections given by the software were plotted against the distance from the soma.

### Mouse brain cortical tissue

Brain cortical tissue samples were obtained from female wild-type (WT) and homozygous HA-tagged CYP46A1 transgenic (C46-HA) mice, previously described and characterized^13^. Briefly, CYP46A1 heterozygous transgenic mice from the sixth generation were inbred for three additional generations in order to obtain the homozygous mice.

### Total cell and tissue extracts

Total cell extracts were obtained by harvesting the cells in PBS and resuspended in lysis buffer (50 mM Tris–HCl, pH 8.0, 150 mM NaCl, 1% Triton-X 100) supplemented with 1 mM dithiothreitol (DTT), 1 mM sodium orthovanadate, 10 mM sodium fluoride and protease inhibitors cocktail 1. Tissue from mouse brain was also homogenized in lysis buffer with a motor-driven Bio-vortexer (NO1083; Biospec Products, Bartlesville, OK, USA). Both cell and tissue samples were incubated at 4 °C, for 30 min. Afterwards, the samples were sonicated four times for 4 seconds each, on ice, followed by centrifugation at 12,000 × *g* for 15 min at 4 °C. The supernatant was recovered and protein concentration was determined by the Bradford method and the extracts were stored at −80 °C until further use.

### Crude synaptosomal fractions

Crude synaptosomal (P2) fractions were obtained from neurons and mouse brain cortex tissue as previously described^46^ with minor modifications. The cell pellet or tissue slices were homogenized in homogenization buffer (4 mM HEPES-NaOH pH 7.3, 0.32 M sucrose) supplemented with 1 mM DTT, 1 mM sodium orthovanadate, 10 mM sodium fluoride and protease inhibitors cocktail 1. Cells were homogenized by 20 passages through a 23 gauge needle attached to a 1-mL syringe and tissue was homogenized with a motor-driven Bio-vortexer (Biospec Products). Samples were centrifuged at 800 × *g* for 10 minutes at 4 °C. The pellet was discarded and supernatant was recovered and subjected to another centrifugation at 9.200 × *g* for 15 minutes at 4 °C. The resulting pellet was resuspended in 100 μl of homogenization buffer and the samples were, once more, centrifuged at 10.200 × *g* at 4 °C. The supernatant was discarded and the pellet resuspended in 100 μl of homogenization buffer. Protein concentration was determined by Bradford method and the extracts were stored at −80 °C until further use.

### Co-immunoprecipitation (Co-IP)

Cortex tissue slices from mouse brain were lysed and homogenized in Co-IP buffer (50 mM Tris-HCl pH 7.5, 150 mM NaCl 1 mM ethylenediaminetetraacetic acid [EDTA], 1% IGEPAL^®^ and 10% glycerol) supplemented with 1 mM sodium orthovanadate, 10 mM sodium fluoride and protease inhibitors cocktail 1. After incubation at 4 °C, for 30 min, samples were centrifuged at 12.000 × *g* for 15 min at 4 °C. The supernatant was recovered and protein concentration was determined by the Bradford method. Samples concentration were adjusted to 2 mg/ml in 500 μl, and pre-cleared with A/G agarose beads saturated with salmon sperm DNA and bovine serum albumin (Upstate, Billerica, MA) for 1 hour at 4 °C. Subsequently, the samples were centrifuged at 2.000 × *g* for 2 min at 4 °C to pellet the beads and recover the cleared supernatant, which was incubated overnight in a rotator at 4 °C with 3 μg of the anti-p-Trk or anti-GGTase-Iβ antibodies. Afterwards, the samples were incubated with A/G agarose beads for 2 hours at 4 °C in a rotator for the binding of the beads to the immunocomplexes. These bead-complexes were pulled-down, washed in Co-IP buffer 3 times, resuspended in Laemmli buffer and heated at 95 °C for 5 min to elute the proteins. Beads were pulled-down and the samples were subsequently subjected to Western blot analysis. In parallel, 50 μg of pre-cleared extract of each tissue sample were used as input. Non-specific IgGs were used as negative controls.

### Western blot

Proteins were subject to SDS-PAGE gels, electroblotted onto Immobilon P membrane (IPVH00010, Merck Millipore) and incubated with specific antibodies. Results were quantified using the Quantity One version densitometry analysis software (Bio-Rad Laboratories Inc., Hercules, CA, USA).

### GGTase-I activity

GGTase-I activity was measured in 96-well plates as previously described^16^,^47^ with minor modifications. Briefly, cells were lysed with lysis buffer (50 mM Tris-HCl pH 7.5, 50 mM MgCl_2_, 50 μM ZnCl_2_, 150 mM KCl, 0.2% octyl-β-D-glucopyranoside and 1% Triton-X 100) supplemented with 1 mM DTT, phosphatase and protease inhibitor cocktail 2. 40 μg of total cell extract were mixed with the reaction buffer (50 mM Tris-HCl pH 7.5, 50 mM MgCl_2_, 50 μM ZnCl_2_, 20 mM KCl, 0.2% octyl-β-D-glucopyranoside) supplemented with 10 μM dansyl-GCVLL, 10 μM GGPP, 1 mM DTT, and phosphatase and protease inhibitor cocktails. Geranylgeranylation of dansyl-GCVLL results in a significant enhancement in fluorescence intensity. The reaction was followed by recording the fluorescence intensity (excitation: 360 ± 40 nM and emission: 528 ± 20 nm) for 10 min using a Synergy 2 Multi-Mode Reader (BioTek Instruments, Inc., Winooski, VT, USA). The initial velocity of the reaction was determined from the slope of the linear stage.

### Total and free cholesterol levels

Total cholesterol was quantified using the Amplex^®^ Red cholesterol assay kit (Invitrogen, Thermo Fischer) according to the manufacturer’s instructions. P2 fraction extracts of mouse brain tissue were placed in a 96-well plate, and the reaction was initiated by adding the Amplex^®^ Red reagent/HRP/cholesterol oxidase/cholesterol esterase working solution to each well. The plate was incubated for 30 min at 37 °C. Fluorescence measurements were performed in a GloMax^®^-Multi Detection System (Promega Corporation, Madison, USA). The protocol to determine free cholesterol in total cell extracts was similar to the one described above, although the working solution did not contain cholesterol esterase. Cholesterol levels were normalized with total protein levels and expressed as μg of cholesterol per mg of protein.

### Expression analysis

Total cell RNA was extracted using Isol-RNA Reagent (5 prime) following manufacturer’s instructions. The RNA was incubated with a recombinant DNase (04716728001, Roche) according to the manufacurer’s instructions to ensure there was no DNA contamination in the RNA samples. Subsequently, cDNA synthesis was performed using the NZY First-Strand cDNA Synthesis Kit (NZYTech, Lda., Lisbon, Portugal) according to manufacturer’s instructions. Real-Time PCR (qPCR) analysis for the rat brain-derived neurotrophic factor (Bdnf); activity-regulated cytoskeleton-associated protein (Arc); glutamate receptor, ionotropic, AMPA 2 (Gria2); glutamate receptor, ionotropic, N-methyl D-aspartate 1 (Grin1); glutamate receptor, ionotropic, N-methyl D-aspartate 2A (Grin2a), glutamate receptor, ionotropic, N-methyl D-aspartate 2B (Grin2b); FBJ murine osteosarcoma viral oncogene homolog (c-fos); glyceraldehyde-3-phosphate dehydrogenase (Gapdh) and the human and rat cholesterol 24-hydroxylase (CYP46A1) was performed using SensiFAST™ SYBR^®^ Hi-ROX Kit (Bioline USA Inc, Taunton, MA, USA) in an ABI 7300 sequence detection system (Applied Biosystems, Foster City, CA, USA) and specific primers (Table 1). The mRNA levels of the genes of interest were normalized to the mRNA levels of Gapdh and expressed as fold changes relative to controls, using the ΔΔCt method.

### 24S-hydroxycholesterol levels

A colorimetric, competitive ELISA kit (ADI-900-210-0001; Enzo Biochem, Inc., Farmingdale, New York) was used for the quantitative determination of 24S-hydroxycholesterol (24OHC) levels in culture media, following the manufacturer’s protocol. Absorbance was measured in the GloMax^®^-Multi Detection System.

### Adenylate kinase cytotoxicity assay

The ToxiLight™ BioAssay Kit (Lonza, Basel, Switzerland) is based on a bioluminescent assay designed to measure the release of the enzyme, adenylate kinase (AK), from damaged cells. Brifely, cell culture supernatants were mixed with AK detection reagent, incubated 5 minutes at room temperature and luminescence was measured using GloMax^®^-Multi Detection System, according to manufacturer’s protocol.

### Lactate dehydrogenase cytotoxicity assay

General cell death was assessed by measuring lactate dehydrogenase (LDH) activity in culture media supernatants, mixed with a solution of lactate (substrate), tetrazolium salt (coloring solution), and NAD (cofactor), according to the manufacturer’s protocol (Roche Applied Science, Indianapolis, IN, USA). Plates were protected from light and incubated for 15  minutes at room temperature. Finally, absorbance was measured at 490 nm, with 620 nm as reference, using a Bio-Rad model 680 microplate reader (Bio-Rad Laboratories, Hercules, CA, USA).

### Statistical analysis

Statistical analysis was performed using the Student’s *t*-test, one-way or the two-way ANOVA tests followed by Tukey multiple comparisons test. All analysis were performed using the GraphPad Prism version 5.01 for Windows, GraphPad Software (La Jolla California USA, www.graphpad.com).

### Ethics statement

Wistar rats were maintained in an animal facility at Instituto de Higiene e Medicina Tropical (IHMT), licensed by the Portuguese Food and Veterinary Directorate General (DGAV). The isolation of cortical neurons was performed by an investigator accredited with FELASA level C. All procedures were performed in accordance with the Portuguese national laws and European guidelines concerning laboratory animals welfare. Regarding WT and C46-HA mice, all procedures were approved by the local ethical committee of Karolinska Institutet, Stockholm (permit numbers: S135-12, S105-11 and S139-10), and performed in accordance with Swedish national and local animal care and use guidelines. All the efforts were made to minimize the suffering of the animals.

## Additional Information

**How to cite this article**: Moutinho, M. *et al*. Neuronal cholesterol metabolism increases dendritic outgrowth and synaptic markers via a concerted action of GGTase-I and Trk. *Sci. Rep.* **6**, 30928; doi: 10.1038/srep30928 (2016).

## Supplementary Material

Supplementary Information

## Figures and Tables

**Table 1:** List of primers used for qPCR.

Gene	Sequence (5′–3′)
*Bdnf*	5′ AGCTGAGCCGTGTGTGACAGT 3′ (fwd)
	5′ ACCCATGGGATTACACTTGG 3′ (rev)
*Arc*	5′ CCCTGCAGCCCAAGTTCAAG 3′ (fwd)
	5′ GAAGGCTCAGCTGCCTGCTC 3′ (rev)
*Gria2*	5′ GACTCTGGCTCCACTAAAGA 3′ (fwd)
	5′ AGTCCTCACAAACACAGAGG 3′ (rev)
*Grin1*	5′ AAGTTCACCTATGACCTTTACC 3′ (fwd)
	5′ CATGACCACCTCACCGAT 3′ (rev)
*Grin2a*	5′ GACAGCAAGAGGAGCAAGTCTC 3′ (fwd)
	5′ CTCAAGGATGACCGAAGATAGC 3′ (rev)
*Grin2b*	5′ GAGTCTGGCTCCACTAAAGA 3′ (fwd)
	5′ AGTCCTCACAAACACAGAGG 3′ (rev)
*c-fos*	5′ AGCATGGGCTCCCCTGTCA 3′ (fwd)
	5′ GAGACCAGAGTGGGCTGCA 3′ (rev)
*Cyp46a1*	5′ GAAGGTCATGCTGGAGGGTATC3′ (fwd)
	5′ CGTAACAGGCGGATGCTCTC 3′ (rev)
*CYP46A1*	5′ TGCGGTCAACGTCTTCCACAA 3′ (fwd)
	5′ CTTGGCCGAAGAGTCTCTCACCA3′ (rev)
*Gapdh*	5′ GGGTGTGAACCACGAGAAAT 3′ (fwd)
	5′ ACTGTGGTCATGAGCCCTTC 3′ (rev)

## References

[b1] Vance J. E. Dysregulation of cholesterol balance in the brain: contribution to neurodegenerative diseases. Dis Model Mech 5, 746–755, 10.1242/dmm.010124 (2012). 23065638 PMC3484857

[b2] Bjorkhem I. Crossing the barrier: oxysterols as cholesterol transporters and metabolic modulators in the brain. J Intern Med 260, 493–508, 10.1111/j.1365-2796.2006.01725.x (2006). 17116000

[b3] Lund E. G., Guileyardo J. M. & Russell D. W. cDNA cloning of cholesterol 24-hydroxylase, a mediator of cholesterol homeostasis in the brain. Proc Natl Acad Sci USA 96, 7238–7243 (1999). 10377398 10.1073/pnas.96.13.7238 PMC22064

[b4] Lund E. G. . Knockout of the cholesterol 24-hydroxylase gene in mice reveals a brain-specific mechanism of cholesterol turnover. J Biol Chem 278, 22980–22988, 10.1074/jbc.M303415200 (2003). 12686551

[b5] Russell D. W., Halford R. W., Ramirez D. M., Shah R. & Kotti T. Cholesterol 24-hydroxylase: an enzyme of cholesterol turnover in the brain. Annu Rev Biochem 78, 1017–1040, 10.1146/annurev.biochem.78.072407.103859 (2009). 19489738 PMC2837268

[b6] Garcia A. N., Muniz M. T., Souza e Silva H. R., da Silva H. A. & Athayde-Junior L. Cyp46 polymorphisms in Alzheimer’s disease: a review. J Mol Neurosci 39, 342–345, 10.1007/s12031-009-9227-2 (2009). 19705089

[b7] Li M. . CYP46A1 intron-2T/C polymorphism and Alzheimer’s disease: an updated meta-analysis of 16 studies including 3,960 cases and 3,828 controls. Neurosci Lett 549, 18–23, 10.1016/j.neulet.2013.06.011 (2013). 23792195

[b8] Fu B. Y. . Cholesterol 24-hydroxylase (CYP46A1) polymorphisms are associated with faster cognitive deterioration in Chinese older persons: a two-year follow up study. Int J Geriatr Psychiatry 24, 921–926, 10.1002/gps.2196 (2009). 19212968

[b9] Lai C. L., Liou L. M., Liu C. K., Yang Y. H. & Lin R. T. Effects of metabolic syndrome, apolipoprotein E, and CYP46 on cognition among Taiwanese Chinese. Kaohsiung J Med Sci 30, 343–349, 10.1016/j.kjms.2014.03.005 (2014). 24924840 PMC11916575

[b10] Hudry E. . Adeno-associated virus gene therapy with cholesterol 24-hydroxylase reduces the amyloid pathology before or after the onset of amyloid plaques in mouse models of Alzheimer’s disease. Mol Ther 18, 44–53, 10.1038/mt.2009.175 (2010). 19654569 PMC2839219

[b11] Djelti F. . CYP46A1 inhibition, brain cholesterol accumulation and neurodegeneration pave the way for Alzheimer’s disease. Brain 138, 2383–2398, 10.1093/brain/awv166 (2015). 26141492

[b12] Kotti T. J., Ramirez D. M., Pfeiffer B. E., Huber K. M. & Russell D. W. Brain cholesterol turnover required for geranylgeraniol production and learning in mice. Proc Natl Acad Sci USA 103, 3869–3874, 10.1073/pnas.0600316103 (2006). 16505352 PMC1450160

[b13] Maioli S. . Is it possible to improve memory function by upregulation of the cholesterol 24S-hydroxylase (CYP46A1) in the brain? PLoS One 8, e68534, 10.1371/journal.pone.0068534 (2013). 23874659 PMC3712995

[b14] Kotti T., Head D. D., McKenna C. E. & Russell D. W. Biphasic requirement for geranylgeraniol in hippocampal long-term potentiation. Proc Natl Acad Sci USA 105, 11394–11399, 10.1073/pnas.0805556105 (2008). 18685105 PMC2516227

[b15] Moutinho M. . Cholesterol 24S-Hydroxylase Overexpression Inhibits the Liver X Receptor (LXR) Pathway by Activating Small Guanosine Triphosphate-Binding Proteins (sGTPases) in Neuronal Cells. Mol Neurobiol 51, 1489–1503, 10.1007/s12035-014-8828-0 (2015). 25084760

[b16] Zhou X. P., Wu K. Y., Liang B., Fu X. Q. & Luo Z. G. TrkB-mediated activation of geranylgeranyltransferase I promotes dendritic morphogenesis. Proc Natl Acad Sci USA 105, 17181–17186, 10.1073/pnas.0800846105 (2008). 18957540 PMC2579398

[b17] Martin M. G. . Cholesterol loss enhances TrkB signaling in hippocampal neurons aging *in vitro*. Mol Biol Cell 19, 2101–2112, 10.1091/mbc.E07-09-0897 (2008). 18287532 PMC2366859

[b18] Marquer C. . Increasing membrane cholesterol of neurons in culture recapitulates Alzheimer’s disease early phenotypes. Mol Neurodegener 9, 60, 10.1186/1750-1326-9-60 (2014). 25524049 PMC4280040

[b19] Nicholson A. M. & Ferreira A. Increased membrane cholesterol might render mature hippocampal neurons more susceptible to beta-amyloid-induced calpain activation and tau toxicity. J Neurosci 29, 4640–4651, 10.1523/JNEUROSCI.0862-09.2009 (2009). 19357288 PMC2705291

[b20] Burlot M. A. . Cholesterol 24-hydroxylase defect is implicated in memory impairments associated with Alzheimer-like Tau pathology. Hum Mol Genet, 10.1093/hmg/ddv268 (2015). 26358780

[b21] Govek E. E., Newey S. E. & Van Aelst L. The role of the Rho GTPases in neuronal development. Genes Dev 19, 1–49, 10.1101/gad.1256405 (2005). 15630019

[b22] Haditsch U. . A central role for the small GTPase Rac1 in hippocampal plasticity and spatial learning and memory. Mol Cell Neurosci 41, 409–419, 10.1016/j.mcn.2009.04.005 (2009). 19394428 PMC2705331

[b23] Tejada-Simon M. V. Modulation of actin dynamics by Rac1 to target cognitive function. J Neurochem 133, 767–779, 10.1111/jnc.13100 (2015). 25818528

[b24] Nakayama A. Y., Harms M. B. & Luo L. Small GTPases Rac and Rho in the maintenance of dendritic spines and branches in hippocampal pyramidal neurons. J Neurosci 20, 5329–5338 (2000). 10884317 10.1523/JNEUROSCI.20-14-05329.2000 PMC6772334

[b25] Yuan M. . Inhibiting geranylgeranyltransferase I activity decreases spine density in central nervous system. Hippocampus 25, 373–384, 10.1002/hipo.22379 (2015). 25330763

[b26] Trovo L., Van Veldhoven P. P., Martin M. G. & Dotti C. G. Sphingomyelin upregulation in mature neurons contributes to TrkB activity by Rac1 endocytosis. J Cell Sci 124, 1308–1315, 10.1242/jcs.078766 (2011). 21444756

[b27] Huang E. J. & Reichardt L. F. Neurotrophins: roles in neuronal development and function. Annu Rev Neurosci 24, 677–736, 10.1146/annurev.neuro.24.1.677 (2001). 11520916 PMC2758233

[b28] Huang E. J. & Reichardt L. F. Trk receptors: roles in neuronal signal transduction. Annu Rev Biochem 72, 609–642, 10.1146/annurev.biochem.72.121801.161629 (2003). 12676795

[b29] Okamoto K., Nagai T., Miyawaki A. & Hayashi Y. Rapid and persistent modulation of actin dynamics regulates postsynaptic reorganization underlying bidirectional plasticity. Nat Neurosci 7, 1104–1112, 10.1038/nn1311 (2004). 15361876

[b30] Okabe A. . Adaptive responses induced by 24S-hydroxycholesterol through liver X receptor pathway reduce 7-ketocholesterol-caused neuronal cell death. Redox Biol 2, 28–35, 10.1016/j.redox.2013.11.007 (2013). 24371802 PMC3871289

[b31] Alexandrov P., Cui J. G., Zhao Y. & Lukiw W. J. 24S-hydroxycholesterol induces inflammatory gene expression in primary human neural cells. Neuroreport 16, 909–913 (2005). 15931060 10.1097/00001756-200506210-00007

[b32] Paul S. M. . The major brain cholesterol metabolite 24(S)-hydroxycholesterol is a potent allosteric modulator of N-methyl-D-aspartate receptors. J Neurosci 33, 17290–17300, 10.1523/JNEUROSCI.2619-13.2013 (2013). 24174662 PMC3812502

[b33] Yamanaka K., Urano Y., Takabe W., Saito Y. & Noguchi N. Induction of apoptosis and necroptosis by 24(S)-hydroxycholesterol is dependent on activity of acyl-CoA:cholesterol acyltransferase 1. Cell Death Dis 5, e990, 10.1038/cddis.2013.524 (2014). 24407243 PMC4040651

[b34] Pfrieger F. W. Cholesterol homeostasis and function in neurons of the central nervous system. Cell Mol Life Sci 60, 1158–1171, 10.1007/s00018-003-3018-7 (2003). 12861382 PMC11138592

[b35] Schulz J. G. . HMG-CoA reductase inhibition causes neurite loss by interfering with geranylgeranylpyrophosphate synthesis. J Neurochem 89, 24–32, 10.1046/j.1471-4159.2003.02305.x (2004). 15030386

[b36] Jin Y. . Atorvastatin enhances neurite outgrowth in cortical neurons *in vitro* via up-regulating the Akt/mTOR and Akt/GSK-3beta signaling pathways. Acta Pharmacol Sin 33, 861–872, 10.1038/aps.2012.59 (2012). 22705730 PMC4011150

[b37] Ko M. . Cholesterol-mediated neurite outgrowth is differently regulated between cortical and hippocampal neurons. J Biol Chem 280, 42759–42765, 10.1074/jbc.M509164200 (2005). 16267051

[b38] Shafaati M. . Enhanced production of 24S-hydroxycholesterol is not sufficient to drive liver X receptor target genes *in vivo*. J Intern Med 270, 377–387, 10.1111/j.1365-2796.2011.02389.x (2011). 21486371

[b39] Hering H., Lin C. C. & Sheng M. Lipid rafts in the maintenance of synapses, dendritic spines, and surface AMPA receptor stability. J Neurosci 23, 3262–3271 (2003). 12716933 10.1523/JNEUROSCI.23-08-03262.2003 PMC6742299

[b40] Brachet A. . LTP-triggered cholesterol redistribution activates Cdc42 and drives AMPA receptor synaptic delivery. J Cell Biol 208, 791–806, 10.1083/jcb.201407122 (2015). 25753037 PMC4362467

[b41] Sodero A. O. . Cholesterol loss during glutamate-mediated excitotoxicity. EMBO J 31, 1764–1773, 10.1038/emboj.2012.31 (2012). 22343944 PMC3321209

[b42] Klein U., Gimpl G. & Fahrenholz F. Alteration of the myometrial plasma membrane cholesterol content with beta-cyclodextrin modulates the binding affinity of the oxytocin receptor. Biochemistry 34, 13784–13793 (1995). 7577971 10.1021/bi00042a009

[b43] Brewer G. J., Torricelli J. R., Evege E. K. & Price P. J. Optimized survival of hippocampal neurons in B27-supplemented Neurobasal, a new serum-free medium combination. J Neurosci Res 35, 567–576, 10.1002/jnr.490350513 (1993). 8377226

[b44] Hitt D. C. . A flow cytometric protocol for titering recombinant adenoviral vectors containing the green fluorescent protein. Mol Biotechnol 14, 197–203, 10.1385/MB:14:3:197 (2000). 10890010

[b45] Gueret V., Negrete-Virgen J. A., Lyddiatt A. & Al-Rubeai M. Rapid titration of adenoviral infectivity by flow cytometry in batch culture of infected HEK293 cells. Cytotechnology 38, 87–97, 10.1023/A:1021106116887 (2002). 19003090 PMC3449930

[b46] Huttner W. B., Schiebler W., Greengard P. & De Camilli P. Synapsin I (protein I), a nerve terminal-specific phosphoprotein. III. Its association with synaptic vesicles studied in a highly purified synaptic vesicle preparation. J Cell Biol 96, 1374–1388 (1983). 6404912 10.1083/jcb.96.5.1374 PMC2112660

[b47] Goossens L., Deweer S., Pommery J., Henichart J. P. & Goossens J. F. Spectroscopic study of fluorescent peptides for prenyl transferase assays. J Pharm Biomed Anal 37, 417–422, 10.1016/j.jpba.2004.11.006 (2005). 15740898

