# Supplementary material for: Neuronal cholesterol metabolism increases dendritic outgrowth and synaptic markers via a concerted action of GGTase-I and Trk
Source: Sci Rep. 2016 Aug 5;6:30928. doi: 10.1038/srep30928 (PMC4974659; doi:10.1038/srep30928)
Supplement: Supplementary Information [file srep30928-s1.pdf]

Supplementary Information

Neuronal cholesterol metabolism increases dendritic outgrowth and synaptic markers via a concerted action of GGTase-I and Trk

Miguel Moutinho[1], Maria João Nunes[1], Jorge C. Correia[2], Maria João Gama[1,3], Margarida Castro-Caldas[1,4], Angel Cedazo-Minguez[5], Cecília M. P. Rodrigues[1,3], Ingemar Björkhem[6], Jorge L Ruas[2], Elsa Rodrigues[1,3]

[1] Research Institute for Medicines (iMed.ULisboa), Faculty of Pharmacy, Universidade de Lisboa, Portugal, Av. Prof. Gama Pinto, 1649-003 Lisboa, Portugal

[2] Department of Physiology and Pharmacology, Molecular and Cellular Exercise Physiology, Karolinska Institutet, 17177 Stockholm, Sweden

[3] Department of Biochemistry and Human Biology, Faculty of Pharmacy, Universidade de Lisboa, Av. Prof. Gama Pinto, 1649-003 Lisboa, Portugal

[4] Department of Life Sciences, Faculty of Science and Technology, Universidade NOVA de Lisboa, 2829-516 Caparica, Portugal

[5] Department of Neurobiology, Care Sciences and Society, Karolinska Institutet-Alzheimer's Disease Research Center, Novum, Stockholm, Sweden

[6]Department of Laboratory Medicine, Division of Clinical Chemistry, Karolinska Institutet, Karolinska University Hospital, Huddinge, Sweden

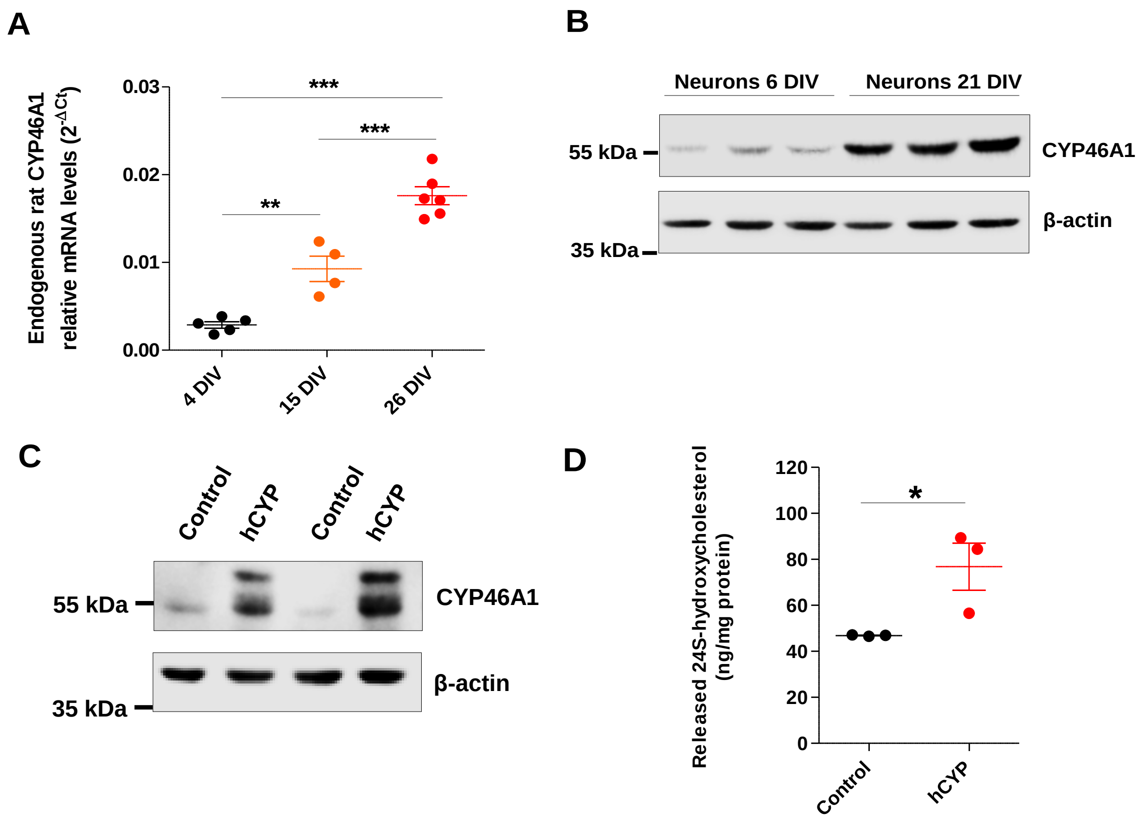

Supp. Fig. 1 – CYP46A1 expression levels in primary cultures of rat cortical neurons. A and B) CYP46A1 endogenous mRNA and protein levels during neuronal maturation *in vitro.* C and D) CYP46A1 protein and 24S-hydroxycholesterol (24OHC) levels in CYP46A1-transfected neurons. A) qPCR analysis of rat CYP46A1 relative mRNA levels in primary cultures of rat cortical neurons 4, 15 and 26 days *in vitro* (4 DIV, 15 DIV and 26 DIV). Data represents mean values ± SEM from at least three independent experiments and is expressed as $2^{-\Delta Ct}$**.** Statistical analysis was performed by one-way ANOVA ($p<0.001$) followed by Tukey post-hoc test (**$p<0.01$ ***$p<0.001$). **B)** Total cell extracts of primary cultures of rat cortical neurons 6 and 21 DIV were subjected to Western Blot analysis for CYP46A1 protein levels. β-actin was used as loading control. **C)** Total cell extracts of primary cultures of 4 DIV rat cortical neurons transfected with pCMV (Control) or pCMV-FLAG-hCYP46A1 (hCYP) and maintained for 48 hours were subjected to Western Blot analysis for CYP46A1 protein levels. β-actin was used as loading control. **D)** Accumulated levels of 24OHC during 48h of transfection (Control and hCYP) were determined in the cell culture media. Data are expressed as ng oxysterol per mg total cell protein. Statistical analysis was performed by Student's *t*-test (*$p<0.05$).

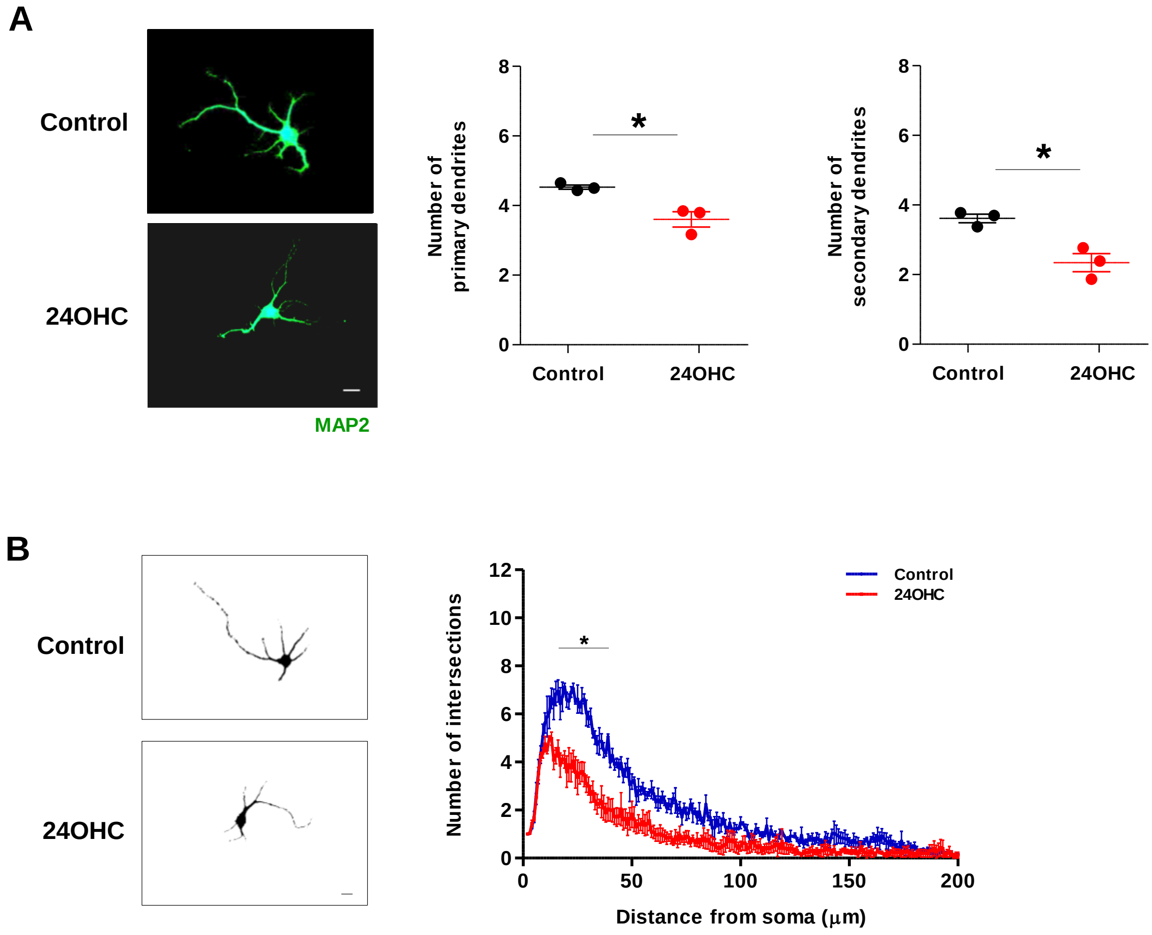

Supp. Fig. 2 – 24S-hydroxycholesterol decreases neuronal dendritic outgrowth. Primary cultures of rat cortical neurons kept for 5 days *in vitro* (5 DIV) were incubated with vehicle (Control) or 10 µM 24S-hydroxycholesterol (24OHC) for 24 hours. Neurons were stained with MAP2 antibodies for dendritic outgrowth analysis at 6 DIV. **A)** Quantitation of primary neuronal dendrites. Images are representative of MAP2 staining (green) in neurons of each experimental condition. Scale bar: 20 µm. Data represents mean values ± SEM from at least three independent experiments and is expressed as number of dendrites. Statistical analysis was performed by Student's *t*-test (**p*<0.05). **B)** Quantitation of dendritic arbor complexity using the Sholl analysis. Images are representative of 6 DIV neurons in each experimental condition. Scale bar: 20 µm. Data represents mean values ± SEM from at least three independent experiments and is plotted as number of intersections versus distance (µm) to soma. Statistical analysis was performed by Student's *t*-test (**p*<0.05).

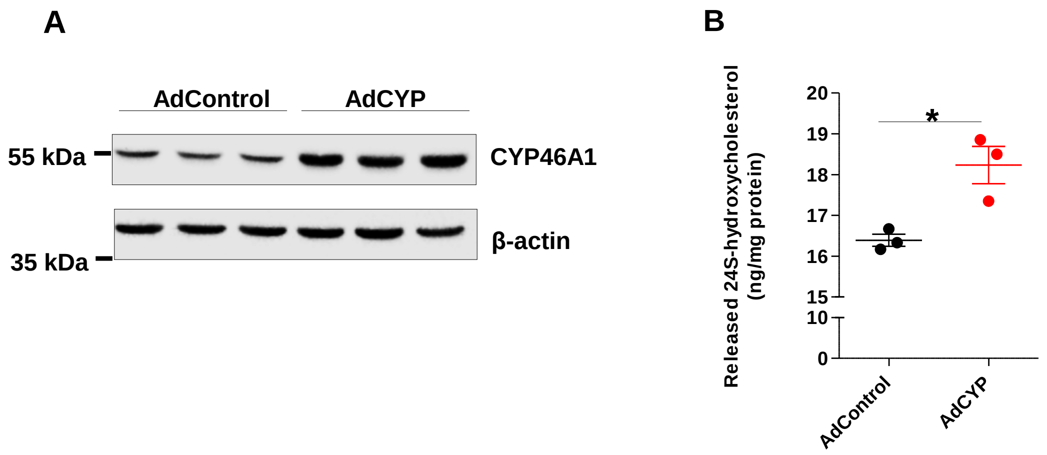

Supp. Fig. 3 - Ectopic expression levels of CYP46A1 protein and 24-hydroxycholesterol levels in primary cultures of rat cortical neurons 19 days *in vitro*. Primary cultures of rat cortical neurons 19 days *in vitro* (19 DIV) were transduced with adenovirus encoding GFP (AdControl) or GFP and FLAG-hCYP46A1 (AdCYP) and maintained for 48 hours **A)** Total cell extracts were subjected to Western Blot analysis for CYP46A1 protein levels. β-actin was used as loading control. **B)** Accumulated levels of 24-hydroxycholesterol during 48h were determined in the cell culture media. Data are expressed as ng oxysterol per mg total cell protein. Statistical analysis was performed by Student's *t*-test (**p*<0.05).

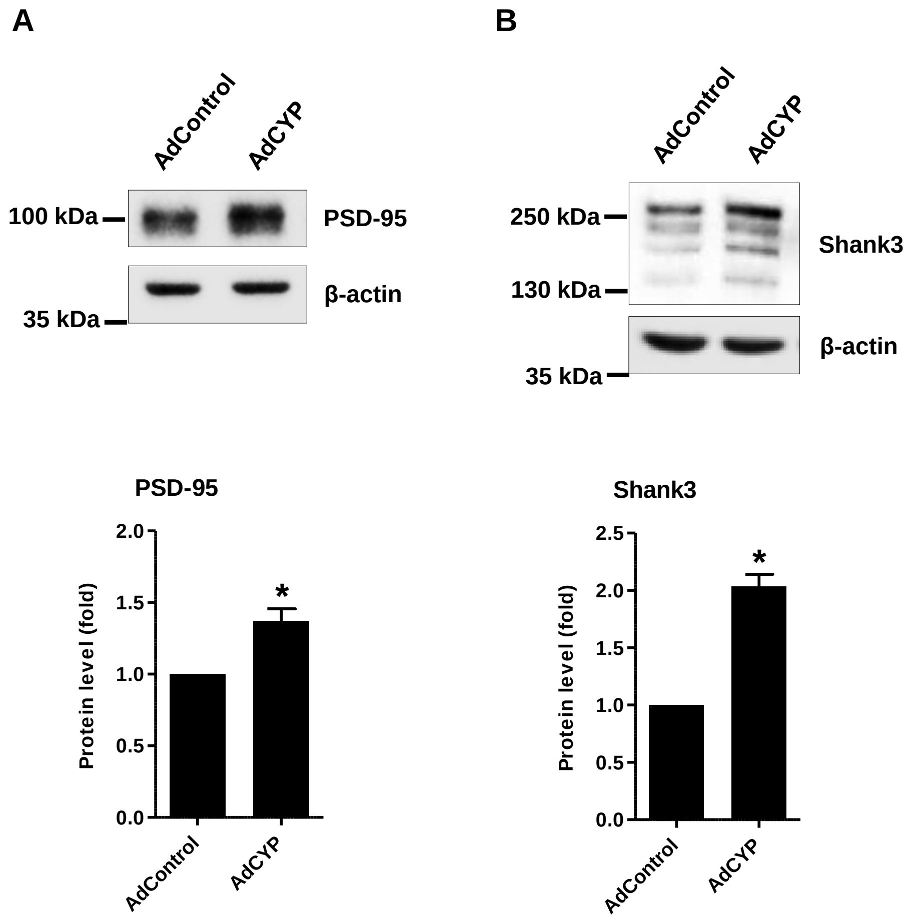

Supp. Fig. 4 - CYP46A1 induces synaptic proteins enrichment in crude synaptossomal fractions (P2). Primary cultures of rat cortical neurons 19 days *in vitro* (19 DIV) were transduced with adenovirus encoding GFP (AdControl) or GFP and FLAG-hCYP46A1 (AdCYP) and maintained for 48 hours. P2 fractions isolated from 21 DIV neurons were subjected to Western Blot analysis for the postsynaptic proteins PSD-95 (A) and Shank3 (B). β-actin was used as loading control. Data represents mean values ± SEM from at least three independent experiments and is expressed as fold change to the control. Statistical analysis was performed by Student's *t*-test (*$p<0.05$).

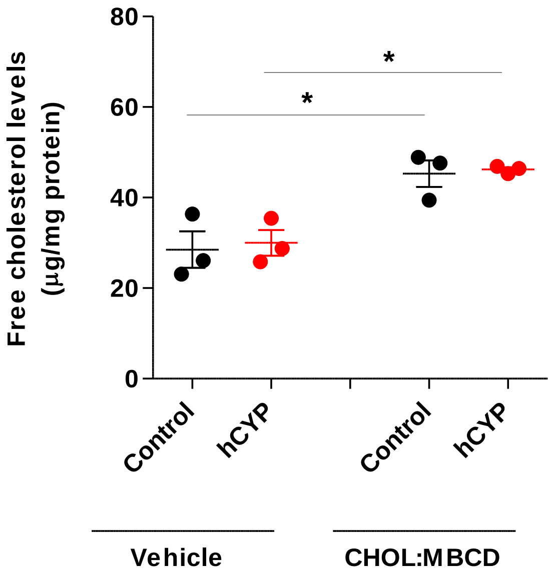

Supp. Fig. 5 - Cholesterol supplementation increases free cholesterol levels in rat cortical neurons. Primary cultures of rat cortical neurons 4 days *in vitro* (4 DIV) were transfected with pCMV (Control) or pCMV-FLAG-hCYP46A1 (hCYP) and maintained for 48 hours. 24 hours after transfection cells were incubated with 10 µM cholesterol (Chol:MBCD). Free cholesterol levels were determined with the Amplex®Red cholesterol determination kit. Data is expressed as µg cholesterol per mg total cell protein and represents mean values ± SEM from at least three independent experiments. Statistical analysis was performed by two-way ANOVA (p=0.924) followed by Tukey post-hoc test (*p<0.05)

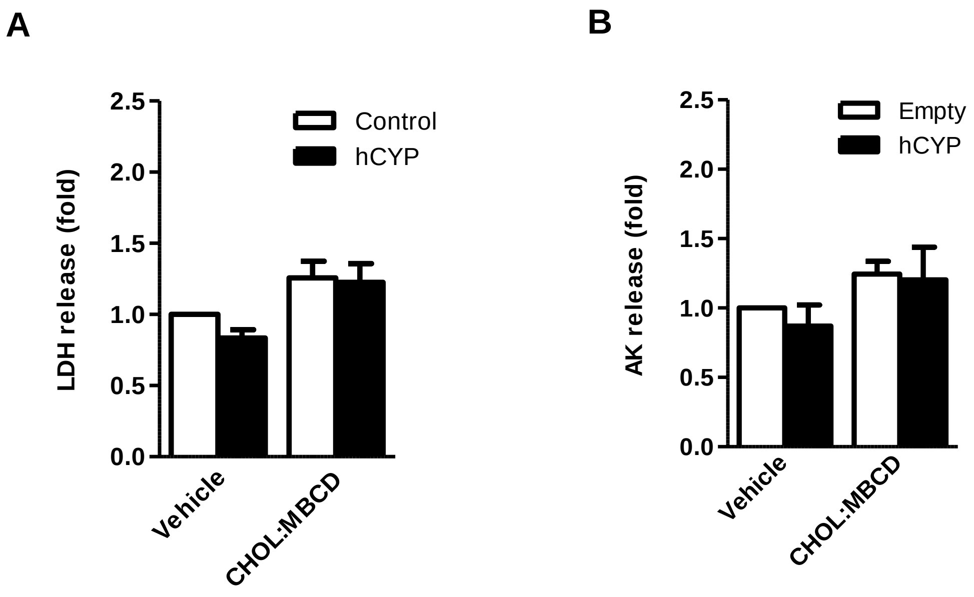

Supp. Fig. 6 - Cholesterol supplementation does not affect cell viability. Primary cultures of rat cortical neurons 4 days *in vitro* (4 DIV) were transfected with pCMV (Control) or pCMV-FLAG-hCYP46A1 (hCYP) and maintained for 48 hours. 24 hours after transfection cells were incubated with 10 µM cholesterol (Chol:MBCD). Cell death was measured by the amount of LDH and AK released to cell culture media in each experimental condition. Data is expressed as fold induction over vehicle-treated Control cells, and represents mean values ± SEM from at least three independent experiments. Statistical analysis was performed by two-way ANOVA (p=0.4894) followed by Tukey post-hoc test.

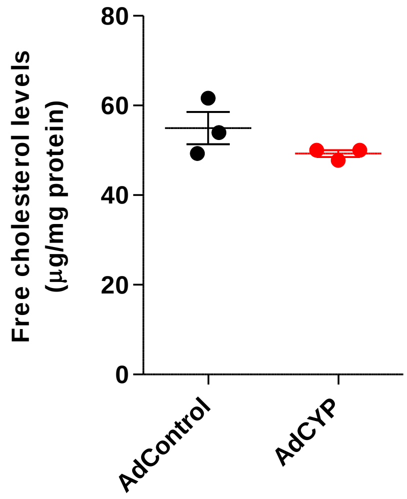

Supp. Fig. 7 - Free cholesterol levels are not affected by CYP46A1 ectopic expression. Primary cultures of rat cortical neurons 19 days *in vitro* (19 DIV) were transduced with adenovirus encoding GFP (AdControl) or GFP and FLAG-hCYP46A1 (AdCYP) and maintained for 48 hours. Free cholesterol levels were determined with the Amplex®Red cholesterol determination kit. Data is expressed as µg cholesterol/ mg total cell protein and represents mean values ± SEM from at least three independent experiments. Statistical analysis was performed by Student's *t*-test.

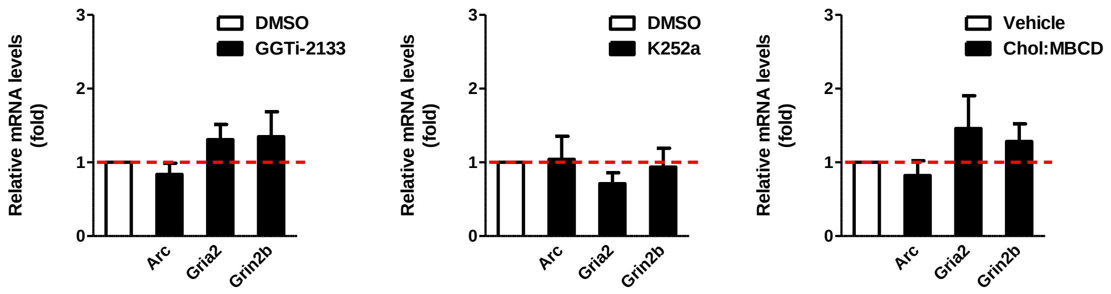

Supp. Fig. 8 - GGTI-2133, K252a and cholesterol supplementation have no effect on Arc, Gria2 and Grin2b mRNA levels. qPCR analysis of Arc, Gria2 and Grin2b mRNA levels in primary cultures of rat cortical neurons 19 days *in vitro* (19 DIV), transduced with adenovirus encoding GFP (AdControl) and maintained for 48 hours. 24 hours after transduction cells were incubated with 100 nM GGTI-2133, 100 nM K252a or 10 µM cholesterol (Chol:MBCD). Data represents mean values ± SEM from at least three independent experiments and is expressed as fold change to the vehicle-treated cells. Statistical analysis was performed by Student's *t*-test.
